# Targeting hypoxic tumor microenvironment in pancreatic cancer

**DOI:** 10.1186/s13045-020-01030-w

**Published:** 2021-01-13

**Authors:** Jinxin Tao, Gang Yang, Wenchuan Zhou, Jiangdong Qiu, Guangyu Chen, Wenhao Luo, Fangyu Zhao, Lei You, Lianfang Zheng, Taiping Zhang, Yupei Zhao

**Affiliations:** 1 Department of General Surgery, Peking Union Medical College Hospital, Chinese Academy of Medical Sciences and Peking Union Medical College, No. 1 Shuaifuyuan, Wangfujing Street, Beijing, 100730 China; 2 Department of Ophthalmology, Xinhua Hospital Affiliated to Shanghai JiaoTong University School of Medicine, Shanghai, 200092 China; 3 Department of Nuclear Medicine, Peking Union Medical College Hospital, Chinese Academy of Medical Sciences and Peking Union Medical College, Beijing, 100730 China; 4 Clinical Immunology Center, Chinese Academy of Medical Sciences and Peking Union Medical College, Beijing, 100730 China

**Keywords:** Hypoxia, Metabolic reprogramming, Redox homeostasis, Autophagy, Stemness, EMT and metastasis, Angiogenesis, Therapeutic resistance, Innovative therapies, Pancreatic cancer

## Abstract

Attributable to its late diagnosis, early metastasis, and poor prognosis, pancreatic cancer remains one of the most lethal diseases worldwide. Unlike other solid tumors, pancreatic cancer harbors ample stromal cells and abundant extracellular matrix but lacks vascularization, resulting in persistent and severe hypoxia within the tumor. Hypoxic microenvironment has extensive effects on biological behaviors or malignant phenotypes of pancreatic cancer, including metabolic reprogramming, cancer stemness, invasion and metastasis, and pathological angiogenesis, which synergistically contribute to development and therapeutic resistance of pancreatic cancer. Through various mechanisms including but not confined to maintenance of redox homeostasis, activation of autophagy, epigenetic regulation, and those induced by hypoxia-inducible factors, intratumoral hypoxia drives the above biological processes in pancreatic cancer. Recognizing the pivotal roles of hypoxia in pancreatic cancer progression and therapies, hypoxia-based antitumoral strategies have been continuously developed over the recent years, some of which have been applied in clinical trials to evaluate their efficacy and safety in combinatory therapies for patients with pancreatic cancer. In this review, we discuss the molecular mechanisms underlying hypoxia-induced aggressive and therapeutically resistant phenotypes in both pancreatic cancerous and stromal cells. Additionally, we focus more on innovative therapies targeting the tumor hypoxic microenvironment itself, which hold great potential to overcome the resistance to chemotherapy and radiotherapy and to enhance antitumor efficacy and reduce toxicity to normal tissues.

## Introduction

Pancreatic cancer (PC) remains a highly fatal malignancy and is the fourth leading cause of cancer-related mortality in both sexes in the US [1], while it is the sixth leading cause of cancer-related mortality in China [2]. Surgical resection represents the only treatment that offers curative potential at present. However, at the time of diagnosis, approximately 80–85% of patients have advanced to either an unresectable or metastatic state, which accounts for the 5-year survival rate of lower than 10% [1]. Even for the small subset (10%) of patients who have the chance to undergo surgery, the prognosis remains poor with only 20% surviving 5 years [3]. Whether receiving surgery or not, chemotherapy and/or radiation therapy are considered primary treatment options. In particular, advancements in adjuvant chemotherapy represented by gemcitabine/capecitabine or mFOLFIRINOX (modified FOLFIRINOX, removing the 5-fluorouracil bolus from the FOLFIRINOX regimen which consists of oxaliplatin, irinotecan, fluorouracil, and leucovorin) currently have improved long-term outcomes in PC patients [4, 5]. However, intrinsic and acquired resistance to chemotherapy remains an intractable problem in PC treatment [6]. Research on new treatments, including immunotherapy and targeted therapy, is ongoing but most of drugs fail to show satisfying outcomes [7, 8]. Therefore, there is an urgent need to develop novel combinatorial therapeutic strategies specifically targeting PC biology.

PC is commonly characterized by numerous and severe hypoxic regions, with a median tissue partial oxygen pressure (pO_2_) of 0–5.3 mmHg (0–0.7%) compared to the adjacent normal pancreas pO_2_ at 24.3–92.7 mmHg (3.2–12.3%) [9]. The hypoxic microenvironment of PC mainly results from desmoplastic fibrotic stroma, rapid proliferation of cancer cells, and poor vascularization, which increase oxygen consumption and compromise oxygen supply [10, 11]. The presence of hypoxic areas within PC is closely correlated with tumor progression and with a poor prognosis compared with that of well-oxygenated tumors, and it is considered one of the independent prognostic factors for PC [12]. The adaptive response to hypoxia primarily mediated by hypoxia-inducible factors (HIFs) confers more aggressive and therapeutically resistant phenotypes in PC cells.

**Fig. 1:**
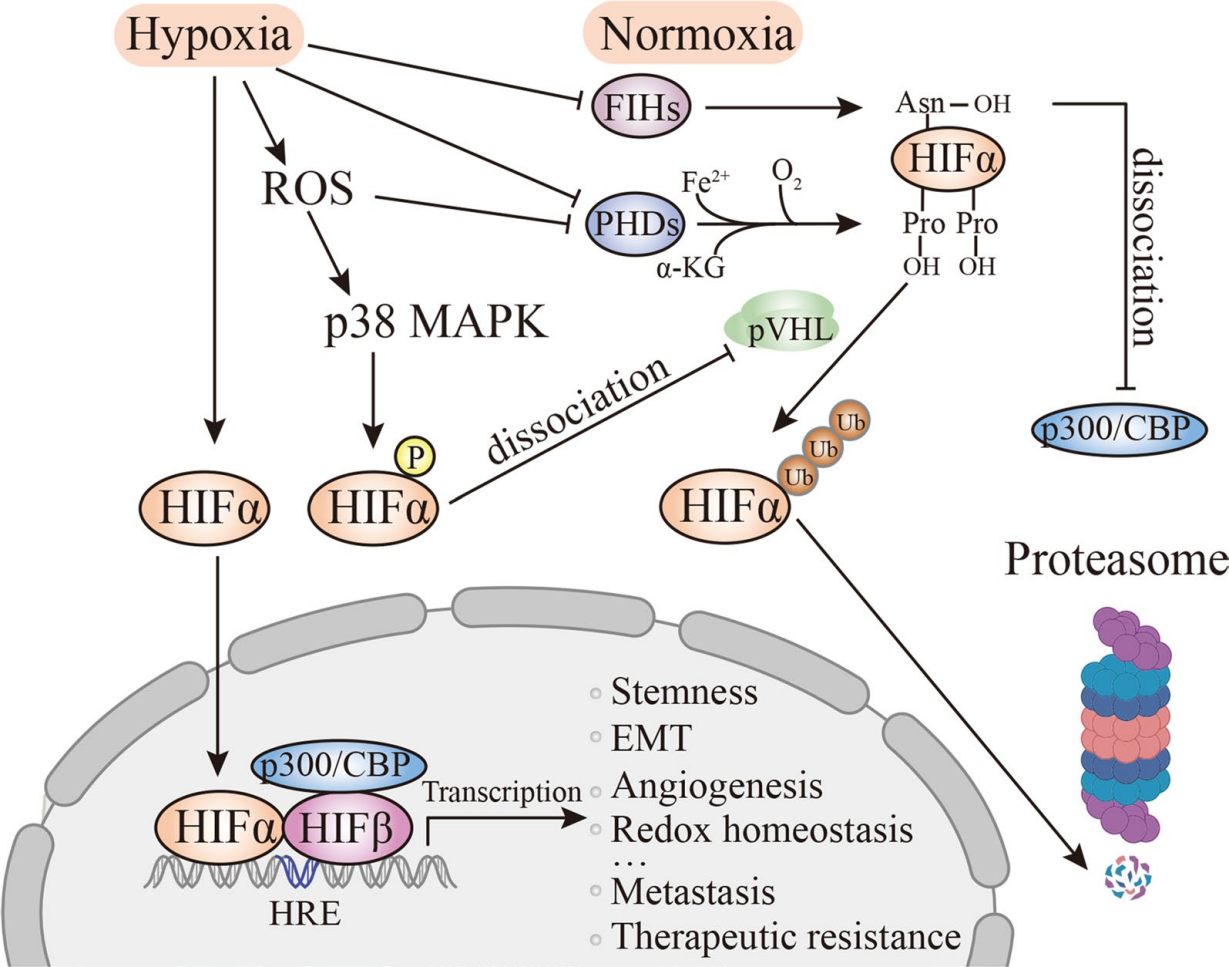
Oxygen-dependent transcriptional regulation of HIFs. Under normoxic conditions, HIF-α protein is continually transcribed and rapidly degraded owing to the posttranslational hydroxylation of highly conserved proline residues by PHDs. Oxygen, Fe^2+^, and α-KG are substrates in this reaction. HIF-α, with hydroxyl group tags, subsequently interacts with the Von Hippel-Lindau protein (pVHL) E3 ubiquitin ligase complex for degradation via the ubiquitin–proteasome pathway. Under hypoxia, the activities of factor inhibiting HIFs (FIHs) and PHDs are suppressed, and thus HIF-α is stabilized and translocated into the nucleus to bind with HIF-β. Inhibition of PHDs and activation of p38 MAPK mediated by ROS are involved in HIF-α stabilization. With the help of transcriptional coactivators such as cyclic adenosine monophosphate response element-binding protein (CBP) and acetyltransferase (p300), the resultant heterodimeric HIF-α/β dimer binds to HREs and transcriptionally activates the targeted genes involved in malignant phenotypes and protumor mechanisms of PC. Abbreviations: Asn, asparagine; Pro, proline

HIFs are heterodimeric transcription factors composed of an unstable α subunit (HIF-α) and a constitutively expressed β subunit (HIF-β) [13, 14]. Figure 1 depicts O_2_-dependent transcriptional regulation of HIFs. Three variants of HIF-α have been discovered, of which HIF-1α is the most studied. HIF-1α and HIF-2α are sensitive to different levels of hypoxia due to their different prolyl hydroxylase sites (Pro564 and Pro402 in HIF-1α, Pro405 and Pro531 in HIF-2α) [15, 16]. HIF-1α accumulates under severe oxygen levels (0–2%) to help cells resist temporary stress [17, 18], whereas HIF-2α exhibits more enduring expression under moderate hypoxia (2–5%) [16, 19]. The functions of HIF-3α primarily rely on the regulation of other HIF complexes [20].

**Fig. 2:**
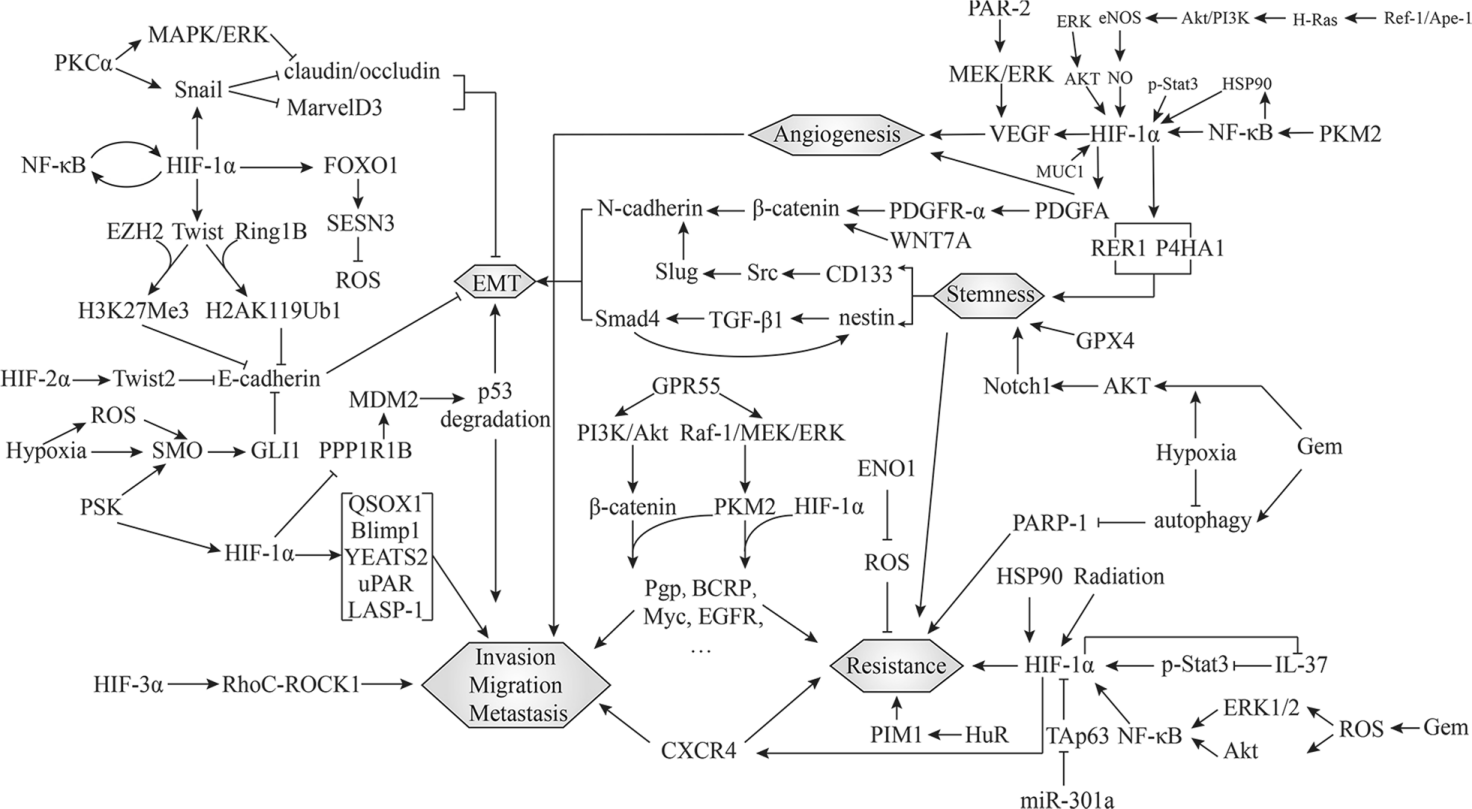
Molecular crosstalk among hypoxia-induced malignant phenotypes in PC. Hexagons represent phenotypes. Arrows indicate positive modulations, while blunt ends indicate negative modulations. Abbreviations: Gem, gemcitabine; QSOX1, quiescin sulfhydryl oxidase 1; LASP-1, LIM and SH3 protein 1

In this review, we summarized the molecular mechanisms underlying hypoxia-mediated malignant phenotypes of PC, which are not confined to HIF-involved modulations. Figure 2 shows a signaling network among hypoxia-induced phenotypes in PC cells. Additionally, we specifically focused on hypoxia-based therapeutic strategies that may have great potential for development into combination therapies for this deadly disease.

### Metabolic reprogramming on glucose under hypoxia

Under the circumstance of hypoxia, PC cells develop an efficient adaptive metabolic response to satisfy high demands for biosynthesis and energy. A major intracellular adaptation to severe hypoxia is the metabolic shift from oxidative phosphorylation to glycolysis, where pyruvate is converted into lactate instead of being oxidized through the tricarboxylic acid (TCA) cycle [21]. Although a low yield of ATP per glucose molecule consumed is generated via glycolytic pathway, it generates intermediates for anabolic reactions, with less reactive oxygen species (ROS) production, which favors rapid cell division [22]. The expression of glycolytic enzymes is increased in PC tissues, as evidenced by proteomic profiling using two-dimensional electrophoresis and liquid chromatography-mass spectrometry/mass spectrometry [23]. Compared with cells exhibiting aerobic glycolysis (Warburg effect), pancreatic hypoxic cells harbor a higher glycolytic potential, leading to a stronger activation of all enzymes and transporters related to glucose uptake and lactic acid formation [24].

Mounting evidence has demonstrated that the underlying mechanisms related to hypoxia play important roles in PC reprogramming glucose metabolism. AMP-activated protein kinase (AMPK), a serine/threonine kinase, is an important cellular energy sensor sustaining energy homeostasis. In an in vitro study, AMPK suppression blocks PC progression and results in a significant decrease in the lactic acid content, ATP content, and the glucose consumption rate, accompanied by down-regulation of glycolytic biomarkers such as pyruvate kinase M2 (PKM2) and hexokinase 2 (HK2) [25]. These observations show that AMPK exerts its protumorigenic functions partially by sustaining glycolytic activity. Nuclear factor of activated T cells 5 (NFAT5), first recognized as a transcription factor that regulates the expression of genes involved in osmotic stress, contributes to glycolytic phenotype rewiring and PC progression via transcription of phosphoglycerate kinase 1 (PGK-1), which is the first enzyme generating ATP in glycolysis [26]. Compared to NFAT5, HIFs are the master transcription factors that regulate transcription of many genes involved in glycolytic enzymes or glucose transporter [27, 28]. Under hypoxia, the expression of HIF-1α is elevated, which increases glycolysis in BxPC-3 cells possibly by upregulating the enzymes related to glycolysis, including pyruvate dehydrogenase kinase-1 (PDK1), lactate dehydrogenase (LDH) A, and PKM2 [29]. Consistently, dominant-negative HIF-1α (dnHIF-1α) abrogates the enhanced transcription of glucose transporter 1 (GLUT1) and aldolase A under hypoxic environment and inhibits the expression of GLUT1 and the glucose uptake in PC tissues and in vivo tumorigenicity [30]. The prolyl 4-hydroxylase subunit alpha 1 (P4HA1) is critically involved in the glycolytic phenotype of pancreatic ductal adenocarcinoma (PDAC) cells, which can be transactivated by HIF-1α under hypoxic condition and further enhances HIF-1α stabilization [31]. Similarly, there is interrelationship between Mucin (MUC)1-HIF-1α oncogenic signaling networks in PC cells [32]. On the one hand, MUC1 may increase the stability of HIF-1α by diminishing the intracellular levels of α-ketoglutarate (α-KG). On the other hand, MUC1 physically interacts with HIF-1α and acetyltransferase (p300) in a hypoxia-dependent manner and facilitates recruitment of HIF-1α and p300 on glycolytic gene promoters. Furthermore, MUC1-regulated stabilization of HIF-1α mediates an increase in pyrimidine biosynthesis [33]. Vascular endothelial growth factor (VEGF) stimulation is known to promote angiogenesis, and it can also enhance glycolysis in PC via upregulation of HIF-1α[34]. AP endonuclease-1/redox effector factor 1 (APE1/Ref-1) is a multifunctional protein with the ability to convert certain oxidized transcription factors to a reduced state, enabling them to bind to and upregulate their target genes [35]. The growth-inhibitory effects of E3330, a small-molecule inhibitor of APE1 redox domain function, are accentuated by hypoxia, indicating an enhanced requirement for APE1 redox function in hypoxia [36]. Blockade of APE1 redox domain with E3330 can inhibit the transcription activating function of HIF-1α and decrease HIF-1α-induced carbonic anhydrase IX (CA9), which help maintain pH homeostasis in cells [35, 37]. Dual-targeting APE1/Ref-1 redox signaling and CA9 activity leads to dramatic enhancement of tumor cell killing in an ex vivo three-dimensional PDAC tumor coculture model even in the presence of the protective environment of the cancer-associated fibroblasts (CAFs) [37].

Oncogenic KRAS mutations have been detected in approximately 93% of PDAC [38]. A role of oncogenic KRAS in reprogramming cancer cell metabolism through activation of glucose metabolism has been documented in various cancer cell lines including pancreatic ones [39]. Introduction of Kras^G12D^ mutation, a mutation frequently observed in PC, could increase mRNA and protein expression levels of RAD51 via MEK/ERK axis. RAD51 further promotes PC glycolysis via upregulation of HIF-1α transcriptional process [40]. Another study demonstrated that KRAS/MEK/ERK signaling exacerbates hypoxia-driven HIF-1α and HIF-2α protein stability in PDAC cells that express activated KRAS, followed by upregulation of downstream HIF-1α-induced effectors such as CA9 and monocarboxylate transporter (MCT) 4, leading to perturbation of pH regulation and metabolic rewiring toward a glycolytic phenotype [41].

PC is characterized by aberrant activation of crucial embryonic signaling pathways such as Wnt signaling [42], which also alters cell metabolic plasticity to support urgent requirements for material and energy. Transcription factor 7-like2/transcription factor 4 (TCF7L2/TCF4) plays a vital role in Wnt/β-catenin signaling pathway. It was identified as a transcriptional factor that was responsible for Egl-9 family hypoxia inducible factor 2 (EGLN2) silencing, which leads to upregulation of HIF-1α and finally affects glycolysis reprogramming [43]. There is a positive-feedback regulation between HIF-2α and β-catenin that HIF-2α/β-catenin complex could upregulate the activity of β-catenin and stabilize and increase transcriptional activity of HIF-2α. HIF-2α promotes the metabolic shift to aerobic glycolysis in PC cells [44].

HIF-1α is involved in certain receptor-mediated glucose metabolism regulation in PC. CX3CL1 is the single cytokine of CX3C group of chemokines and exerts its biological function via binding to its specific receptor, CX3CR1. CX3CL1/CX3CR1 signaling pathways could upregulate the expression of HIF-1α by PI3K/Akt and MAPK pathways and modify glucose metabolism in a HIF-1α-dependent manner in PC cells [45]. When insulin receptor (IR) and IGF-1 receptor (IGF1R) of PC cell are activated, the message passes along PI3K/Akt and MEK/ERK signaling cascades, and subsequently HIF-1α expression is elevated. HIF-1α further increases caveolin-1 (cav-1) which conversely augments IR/IGF1R activities. The feed-forward loop composed of IR/IGF1R, HIF-1α and cav-1 facilitates cell growth and glycolysis [46]. Lumican is widely expressed in PDAC and surrounding stroma. Extracellular lumican reduces expression and phosphorylation of EGFR by enhancing its dimerization and internalization, leading to attenuation of downstream PI3K/Akt pathway, and thus reduces HIF-1α expression and inhibits glycolytic metabolism [47].

Epigenetic research has provided a more comprehensive approach for a better understanding of reprogramming glucose metabolism of PC. Ubiquitin like with plant homeodomain (PHD) and ring finger domains 1 (UHRF1) is a chromatin modifier and mediates gene silencing through DNA methylation and heterochromatin formation [48, 49]. UHRF1 silences expression of its downstream target, sirtuin (SIRT) 4, which suppresses HIF-1α and negatively regulates aerobic glycolysis. Therefore, UHRF1/SIRT4 axis increases expression of HIF-1α-targeted glycolytic genes and enhances aerobic glycolysis in PC [10]. The histone demethylase lysine specific demethylase 1 (LSD1), involved in the epigenetic regulation of gene transcription, is significantly increased in PC tissue samples. LSD1 regulates HIF-1α protein stability in an acetylation-dependent manner through recruitment of HDAC2, which prevents HIF-1α from subsequent acetylation-dependent degradation and thus stabilizes HIF-1α protein. Interestingly, elevated HIF-1α protein level positively correlates with mRNA and protein level of LSD1. These observations suggest that LSD1 and HIF-1α regulate each other and form a positive feedback loop in sustaining the hypoxic microenvironment and shifting in glucose metabolism transformation [12]. Lactate dehydrogenase (LDH) is the key glycolytic enzyme responsible for the interconversion between pyruvate and lactate, including two types of subunits, M subunit (muscle-type, LDHA) and H subunit (heart-type, LDHB). HIF-1α/-2α could activate LDHA expression in human PC cells by binding to LDHA at 89 bp under hypoxia. Knockdown of endogenous HIF-1α and HIF-2α reduces the LDHA expression even in hypoxic condition, accompanied with a significant decrease in glucose utilization and lactate production [50]. Compared to the elevated LDHA level [10, 29, 40, 50], the expression of LDHB is suppressed and even lost in PC tissues due to hypermethylation of its promoter. Decreased expression of LDHB leads to glycolytic transition, while restored its expression leads to reduced LDH activity and lactate production but increases intracellular ATP concentration [51]. In addition to DNA and histone modifications, miRNAs also participate in epigenetics regulation on glucose metabolism transition [52]. For example, miR-124 inhibits the expression level of HIF-1α and LDHA by targeting MCT1, an important carrier in lactate transport, and thus suppresses the glycolytic activity of PANC-1 cells [53].

### Metabolic reprogramming on glutamine under hypoxia

Apart from activating glucose uptake and glycolysis, PDAC cells also enhance utilization of the amino acid glutamine (Gln) as a carbon fuel source for survival in hypoxic environment [54, 55]. Gln is initially deaminated to glutamate (Glu) by the mitochondrial glutaminase (GLS) [56]. The isoform 2 (GLS2) is preferentially expressed in hypoxic areas of PC, suggesting that glutamate generation in pancreatic hypoxic cells is essentially dependent on GLS2 but not GLS [24]. Classically, in most cells, Glu is subsequently converted to α-KG by glutamate dehydrogenase (GDH), and then α-KG is incorporated into the TCA cycle [56]. However, PDAC cells catalyze oxaloacetate (OAA) and Glu to aspartate and α-KG via the mitochondrial aspartate transaminase (GOT2), and then aspartate is metabolized to OAA in the cytoplasm by cytoplasmic aspartate transaminase (GOT1) [57]. OAA is finally converted to malate and pyruvate in the cytoplasm in turn via malate dehydrogenase 1 (MDH1) and malic enzyme 1 (ME1), respectively [58]. This process is termed noncanonical Gln metabolism of PDAC, resulting from increased GOT1 expression but decreased GDH level in PC cells [57]. PDAC cells are especially dependent on Gln to sustain a rapid proliferation rate [57].

Loss of heterozygosity for Kras^G12D^ (Kras^G12D^-LOH) substantially increases non-canonical Gln metabolism in PDAC cells, accompanied by enhanced expression of regulated in DNA damage and development 1 (REDD1), which is a stress-response gene expressed in response to hypoxia and nutrient deprivation. REDD1 knockdown efficiently inhibits Kras^G12D^-LOH-induced upregulation of Gln metabolism, suggesting the essential role of REDD1 as a downstream target of Kras^G12D^-LOH in PDAC Gln metabolism [59]. Hypoxia-activated expression of the SLC1A5 variant is HIF-2α dependent. As a mitochondrial Gln transporter, it is localized to the inner mitochondrial membrane via its N-terminal mitochondrial targeting sequence and drives a metabolic switch by enhancing Gln metabolism in PC cell [60]. Unlike PDAC cell lines, CAFs are dependent on Gln rather than glucose for viability. Moreover, distinct from the GOT-mediated Gln metabolic pathway in PDAC cells, Gln is the preferred metabolite for the TCA cycle in CAFs through a glutamate dehydrogenase (GLUD1)-mediated utilization of Gln [61]. Hypoxia within PDAC benefits a shift to both glucose- and Gln-dependent anaerobic metabolic pathways. Additionally, hexosamine biosynthetic (HBP) pathway that requires both metabolized glucose and Gln is also activated, enabling O-GlcNAC modifications of protumoral proteins [24]. GLS inhibition effectively reduces PDAC growth by specifically targeting proliferating cancer cells but have no effect on hypoxic, noncycling cells. These hypoxic tumor cells surviving from GLS inhibition are reliant on glycolysis and glycogen synthesis. Based on these observations, it was proposed that rapidly dividing cells use Gln, while hypoxic cells that are slowly dividing metabolize glucose [62]. But this hypothesis seems to be paradox with the findings that Gln metabolization is necessary to support hypoxic PC cells for viability maintenance, proliferation, and colony formation capacities [24]. Further studies should be conducted to generate a deeper understanding of the ambiguous role of Gln metabolic pathway in hypoxic PC cells.

Reprogramming of glutamine metabolism also contributes to lipogenesis and proliferation in carcinoma cells due to reductive glutamine metabolism by isocitrate dehydrogenase 1 (IDH1) under hypoxia, which is another non-canonical glutaminolysis pathway in the cytoplasm [63, 64]. The metabolic shift from TCA cycle to the above IDH-mediated lipogenesis correlates with HIF-1-induced decreased activity of α-ketoglutarate dehydrogenase (α-KGDH) [65]. Moreover, the elevated expression of short chain acyl-CoA synthetases 2 (ACSS2) under hypoxia facilitates acetate uptake for synthesis of fatty acid and membrane phospholipids in several carcinoma cells including PC cells [66, 67]. Apart from leading to lipid synthesis, hypoxia also suppresses lipolysis/β-oxidation via HIF-1α-induced downregulation of medium- and long-chain acyl-CoA dehydrogenase, and accumulation of lipid droplets promotes cancer progression [68]. Furthermore, hypoxia could mediate the uptake of monounsaturated fatty acids from extracellular lysophospholipids in human pancreatic duct cells transfected with oncogenic Kras^G12D^ [69]. Overall, compared to glucose or glutamine metabolism, the research on hypoxia-driven metabolic reprogramming was less focused on lipid metabolism in PC, which awaits further study.

### Redox homeostasis under hypoxia

Extensive research has shown that hypoxia increases the generation of reactive oxygen species (ROS) in PC cells as well as stromal cells. Hypoxia correlates with a state of oxidative stress, and ROS induction is one of the most common regulatory mechanisms under hypoxic conditions [70]. A modest amount of ROS below a threshold level triggers tumor cell survival and proliferation, for example, through the activation of the ERK1/2 and PI3K/Akt pathways [71], whereas an excessive amount of ROS leads to a deleterious process by increasing lipid peroxidation, protein oxidation, and DNA lesion formation [72]. Endoplasmic reticulum oxidoreductase 1 (ERO1L) level is upregulated by hypoxia, and it significantly increases ROS level. Treatment with glutathione (GSH) inhibitors results in a significant increase in ROS levels and a transient upregulation in glycolysis in PDAC cells. However, this treatment does not augment ROS-mediated long-term promotion of cell proliferation upon hypoxia-activated ERO1L overexpression, suggesting that high ROS levels may induce an oxidative stress in PDAC cells and ultimately decrease cell viability [73]. ROS generation is induced in a HIF-1α-independent manner under hypoxic conditions [74], but increased ROS is responsible for HIF-1α stabilization [75, 76]. One of the mechanisms underlying HIF-1α stabilization is ROS-mediated inhibition of prolyl hydroxylases (PHDs) by oxidation of their catalytic Fe^2+^ [77–79]. Moreover, ROS-mediated activation of p38 MAPK is involved in stabilizing HIF-1α via phosphorylation of HIF-1α which inhibits its ubiquitination and degradation [79, 80]. However, high levels of hydrogen peroxide (H_2_O_2_) inhibit HIF-1α protein accumulation [81]. High-dose P-AscH^−^ administered intravenously serves as a prodrug for the selective delivery of H_2_O_2_ to cancer cells and induces cytotoxicity and oxidative stress. This cytotoxicity is correlated with increased degradation of HIF-1α in an H_2_O_2_-dependent manner [82].

The abnormal vasculature of PC greatly contributes to intermittent opening of blood vessels. This physiological characteristic as well as a dose of radiation could lead to reoxygenation of hypoxic cancer cells [83, 84]. The phenomenon of hypoxia and reoxygenation (H/R) commonly induces ROS generation [85]. H/R condition triggers increased generation of mitochondrial ROS and lipid peroxidation products in KP4, a human PC-derived cell line. H/R-induced cellular oxidative injuries can be efficiently prevented by mitochondria-localized MnSOD, a superoxide-scavenging enzyme [86]. However, nicotinamide adenine dinucleotide phosphate (NADPH) oxidase-dependent generation of ROS acts downstream of Rac GTPase 1 (Rac1), enhancing the invasive capacity of human PC PANC-1 cell line under H/R conditions [87]. Further research is needed to figure out whether the dual roles of ROS are associated with their sources.

**Fig. 3:**
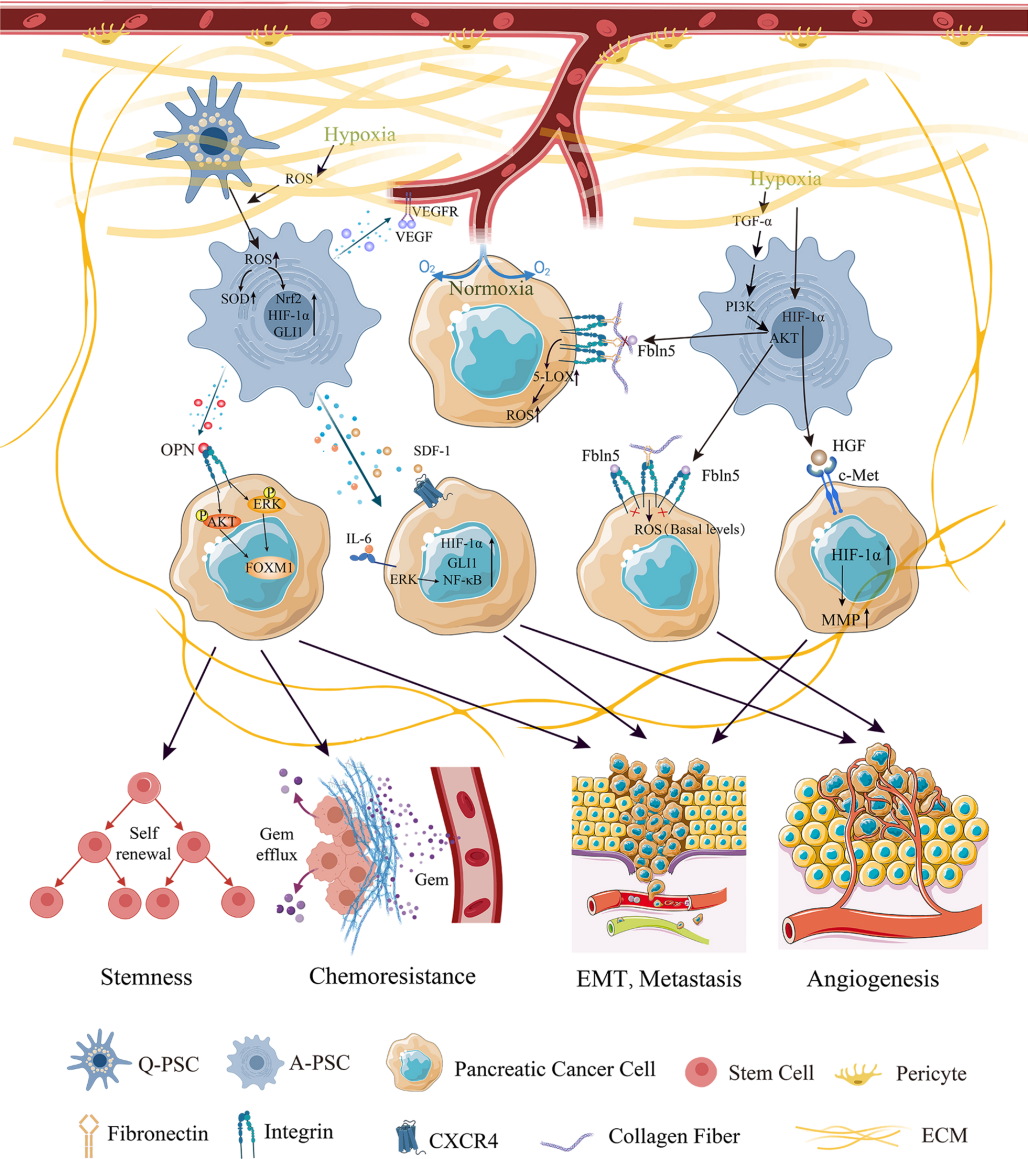
Effects of microenvironmental remodeling on PC progression under hypoxia. Hypoxia-driven ROS production activates PSCs to secrete various soluble factors, favoring the malignant phenotypes of PC. Hypoxia-stimulated TGF-α signaling induces Fbln5 expression through a PI3K/AKT-dependent mechanism in A-PSC, and Fbln5 competes with fibronectin for integrin binding and reduces ROS production. Upward arrows indicate upregulation of expression, and other arrows indicate positive modulations or release of molecules. Abbreviations: A-PSC, activated pancreatic stellate cells; Gem, gemcitabine; Q-PSC, quiescent pancreatic stellate cells

In addition to PC cells, hypoxia is a contributing factor for ROS induction and activation in stromal cells such as pancreatic stellate cells (PSCs) whose roles are linked to their function of paracrine. The levels of ROS produced by PSCs are significantly increased under hypoxia compared to those under normoxia [70, 88]. Hypoxia induces PSC activation and increases both the expression and secretion of osteopontin (OPN) in PSCs rather than in PCCs in a ROS-dependent manner. More importantly, paracrine OPN signaling contributes to epithelial-mesenchymal transition (EMT) and stemness by activating the integrin αvβ3-Akt/Erk-FOXM1 cascade in BxPC-3 cell line [70]. Activated PSCs cultured under hypoxia exploit their hypoxia-driven oxidative stress to secrete soluble factors, including IL-6, VEGF-A, and stromal cell-derived factor-1 (SDF-1), favoring angiogenic and inflammatory responses and invasion during PC progression. Increased generation of hypoxic ROS also promotes HIF-1α stabilization and GLI1 upregulation in both PSCs and PCCs cultured with PSC conditioned media. These hypoxia-driven ROS-induced effects can be suppressed by antioxidant α-Mangostin [88]. Figure 3 shows crosstalk between PC cells and PSCs under hypoxic conditions.

PC develops various mechanisms to eliminate excess ROS generated during hypoxia. The aberrant deposition of extracellular matrix (ECM) proteins is a defining characteristic of PC. ECM signaling can exert antitumorigenic effects due to generation of cytotoxic levels of ROS via fibronectin-mediated stimulation of α5β1 integrin [89, 90]. Fibulin-5 (Fbln5), a matricellular protein typically expressed by stromal cells and acts as a tumor promoter by blocking fibronectin-mediated integrin signaling and directly limiting ECM-driven ROS generation [91]. Hypoxia-stimulated TGF-α signaling can induce fibulin-5 expression through a PI3K/AKT-dependent mechanism in fibroblasts, which protects PC from oxidative stress damage and promotes its progression [89] (Fig. 3). PSCs undergo a pro-oxidant condition under hypoxia, with the oxidation of proteins and lipids increased. However, mitochondrial ROS increase slightly, and no increase in cytosolic ROS is detected. Consistent with these observations, PSCs exhibit activation of major antioxidant mechanisms to counteract the hypoxia-triggered pro-oxidative status, including augmented SOD1/2 activity, increased phosphorylation of transcription factor Nrf2, and overexpressed Nrf2-regulated antioxidant enzymes, such as the catalytic subunit of glutamate-cysteine ligase (GClc), catalase, NAD(P)H quinone oxidoreductase 1 (NQO1), and heme oxygenase-1 (HO-1) [92]. In PDAC cells, hypoxia also upregulates the expression of HO-1, which reduces intracellular ROS levels and provides a survival advantage to cancer cells [93]. HIF-1α has been demonstrated to inhibit the production of a certain amount of mitochondrial ROS under hypoxia, for example, through regulation of cytochrome c oxidase and PDK1 [94, 95]. In BxPC-3 and PANC-1 cells, HIF-1 significantly eliminates intracellular ROS uncoupled from the respiratory chain and NADPH oxidase, indicating that HIF-1 eliminates ROS independent of the mitochondrial respiratory chain. HIF-1/FOXO1/SESN3 pathway probably mediates the role of HIF-1 in ROS clearance [96].

### Autophagy under hypoxia

Autophagy is an evolutionarily preserved degradation process of cytoplasmic cellular constituents, associated with cell response to stresses such as hypoxia in cancer microenvironment [97]. As a key component and a marker of autophagy, LC3 expression in PC tissue significantly correlates with a poor outcome [98]. The RNA splicing protein polypyrimidine tract-binding protein 3 (PTBP3) is overexpressed in PDAC and promotes autophagy in response to hypoxic stress. It upregulates the expression of ATG12 post-transcriptionally by selectively binding to CU-rich elements in the 3′-untranslated regions (3′-UTR) of ATG12, thereby contributing to hypoxia-induced autophagy and gemcitabine resistance [99]. Moreover, hypoxia-mediated ROS production inhibits Akt/mTORC1 pathway to induce autophagy in PC [100].

Hypoxia can abrogate the effect of tumor suppressors to facilitate PC progression by autophagy. Hypoxia stimulates HIF-1α and AMPK activation, which induces autophagy of PSCs and subsequent degradation of lumican [101]. Lumican is identified as an inhibitor of cancer development, involved in the regulation of ECM and ECM-cell interactions [102]. Its synthesis is also inhibited by the blockage of Akt/mTOR signaling pathway mediated by hypoxia-activated AMPK [101]. KAI1 is an important member of metastasis suppressor gene family, but hypoxia protects MiaPaCa-2 cells from KAI1-induced proliferation inhibition and apoptosis by high levels of autophagy induction. Although hypoxia-induced autophagy results in oncogenic protein MUC4 degradation, PC cell growth and viability are enhanced. It is possible that the increased expression of MUC1 under hypoxia is sufficient to compensate for MUC4 downregulation [100].

Upregulation of autophagy-inhibitory factors impairs PC development. The metastasis suppressor, N-myc downstream regulator gene 1 (NDRG1), inhibits basal and hypoxia-induced autophagic activity through a dual acting mechanism, namely both the autophagic degradation and autolysosome formation. Moreover, the NDRG1-inducer sensitizes PANC-1 cells to lysosomal membrane permeabilization (LMP) and exerts an anti-cancer activity [103]. MiR-138-5p is among the most downregulated miRNA in PC cells cultured under hypoxia and inhibits autophagy and PC growth in vivo [104]. Mechanistically, it suppresses autophagy of PANC-1 cells in a manner independent of the typical autophagy signaling pathway. Instead, miR-138-5p specifically targets the 3′-UTR region of autophagy-related gene SIRT1 and reduces its expression to inhibit autophagy via SIRT1/FOXO1/Rab7 axis. Exceptionally, in PaCa cells, basal autophagic activity is relatively high, whereas hypoxia-induced autophagy, is only weak or undetectable [105]. Thus, it is essential to choose a proper cell line to study autophagy in PC.

Hypoxia-induced autophagy seems to play dual roles in PC cell death. Protein kinase C-delta (PKCδ) constitutively inhibits autophagy by inducing expression of tissue transglutaminase (TG2). Suppression of PKCδ/TG2 signaling would lead to significant autophagic cell death mediated by Beclin 1 in PC [98]. Stress response protein Nupr1 is systematically overexpressed when PDAC-derived cells submitted to hypoxia. It promotes survival of PC cells by inhibiting autophagy in a caspase-independent manner. The inhibitory effects on autophagy of Nupr1 are mediated by its downstream Aurora kinase A (AURKA) which prevents DNA damage and autophagic-cell death in hypoxic microenvironment [106]. However, it was also reported that autophagy blockage in a hypoxic state induces a remarkable cytotoxicity and enhances apoptosis in a panel of PC cell lines, whether non-metastatic or metastatic, with an AMPK inhibitor added or not [107, 108]. The roles of autophagy on PC cell death under hypoxic condition await further research.

### Maintenance of stemness under hypoxia

Hypoxic areas within tumors provide well-established niches for cancer cells to acquire and maintain stem-like properties, referred to as the cancer stem cell (CSC) phenotype, such as self-renewal, sphere-forming and metastatic ability, and an undifferentiated state [109, 110]. Pancreatic CSCs harbor high heterogeneity in terms of their surface and intracellular markers as well as their response to chemotherapy, radiation, and hypoxia [111]. Although many pancreatic CSC markers have been reported, such as CD24, CD44, epithelial surface antigen (ESA) [112], CD133 [109], CD184 (C-X-C chemokine receptor type 4, CXCR4), side population (SP) [113], nestin [114], and c-Met [115], relatively few studies have determined the underlying mechanisms regarding how these CSC markers regulate the stemness or tumorigenicity of PC. CD133 enriched in Capan1M9 cell line encourages cell migratory ability and EMT phenomenon by increasing expression and transcriptional activity of HIF-1α under hypoxia [116]. However, mechanisms regarding how CD133 modulates HIF-1α expression are not fully known. Expression of another pancreatic CSC marker nestin is necessary to drive EMT and to promote cell migration and invasion in PDAC. Hypoxia induces nestin expression in PDAC cells via the TGF-β1/Smad4 pathway. More importantly, elevated nestin expression promotes a positive feedback constitutive activation of TGF-β1/Smad signaling by increasing the expression of TGF-β1, TβR1, and TβR2 in nestin-positive PDAC cells [117].

HIF-dependent transcriptional activity is a key contributor to PC stemness [118, 119]. CD133^+^ cells colocalize to the hypoxic areas within PC and have an increased HIF-1α activity [120]. A subpopulation of human PC cell lines was observed to upregulate CD133 and increase invasiveness in a predominantly HIF-1α-dependent manner under hypoxia [110]. Under hypoxic condition, the percentage of CD133^+^ PC cells as well as markers for stemness including Sox2, Bmi1, Lin28, and Nanog were reduced by retention in endoplasmic reticulum 1 (RER1) knockdown but were increased by RER1 overexpression, suggesting hypoxia-induced stemness may be mediated by RER1. HIF-1α acts as an upstream positive regulator of RER1 and thus participates in hypoxia-induced pancreatic CSC phenotype [121]. Similarly, HIF-1α increases PDAC stemness marker expression indirectly through induction of P4HA1 expression [31]. Triptolide, an anticancer compound, significantly decreases HIF-1α transcriptional activity via depletion of its coactivator p300 which involves the assembly of the transcription complex of HIF-1α, leading to the decreased population of stem cells in PC [122]. Additionally, the increases of CD133^+^ cells and stemness-associated proteins Nanog and Oct-4 are closely associated with the overexpression of HIF-2α, suggesting that HIF-2α may be also involved in maintenance of PC stemness [44].

Hypoxia-induced autophagy has a potential role in the induction and maintenance of PC stemness. Intermittent hypoxia maintains stem cell-like properties and upregulates the expression of CSC signature markers such as CD133, Oct-4, and Sox-2, as well as enhances HIF-1α-induced autophagy in PANC-1 cells [123]. It was further demonstrated that HIF-1α-mediated autophagy is critical for the conversion of non-stem PC cells into CD133^+^ stem-like cells under intermittent hypoxia [124]. Physiologically elevated levels of autophagy enable the survival of pancreatic CSCs in a hypoxic microenvironment. Interference of autophagy with its inhibitors or activators resensitizes PC stem-like cells to apoptosis and diminishes the CSC phenotype and expression of CSC-related genes in vivo [125].

Maintenance of oxidative homeostasis regulates PC stemness by balancing ROS levels. Hypoxia-driven, ROS-induced PSC activation increases the expression and secretion of OPN, which promotes pancreatic CSC-like properties by activating the integrin αvβ3-Akt/Erk-FOXM1 pathway in a paracrine manner [70]. Inhibition of HO-1, an Nrf2-regulated antioxidant enzyme, under hypoxia not only increases ROS production but also reduces the expression of stemness markers CD133 and CD44 in Capan-1 and CD18/HPAF cell lines, indicating that hypoxia-induced PC stemness is probably mediated by HO-1 via its capability of reducing intracellular ROS levels [93]. Compared with non-stem PANC-1 cells, PANC-1 CSCs express a relative high level of endogenous GPX4, which regulates oxidative homeostasis and sustains the stemness phenotype in PANC-1 CSCs [126]. Indeed, hypoxia-triggered CD133^+^ PC cells present lower production of ROS compared to CD133^−^ cells, and they even show little or no ROS accumulation following treatment with cytotoxic chemotherapeutic drugs that classically induce ROS production. Low mitochondrial complex I and complex IV activity in CD133^+^ PC cells may protect them from ROS accumulation [120].

### EMT, invasion, migration, and metastasis under hypoxia

The oxygen concentration in the microenvironment is a dynamic switch for plasticity regulation in cells [127]. Intratumoral hypoxia induces epithelial-mesenchymal transition (EMT) transformation in PC cells, while exposure to normoxia or hyperoxia causes a reversal of EMT [128]. A considerable amount of literature has previously indicated that HIF-1α [129] and NF-κB [130, 131] plays critical roles in hypoxia-induced EMT. Hypoxia or overexpression of HIF-1α promotes EMT in PC cells, which is largely dependent on NF-κB activity [132]. Furthermore, the expression of HIF-1α protein is inhibited by NF-κB(p65) siRNA, while the overexpression of NF-κB(p65) protein and NF-κB DNA binding activity are inhibited by HIF-1α siRNA, indicating that HIF-1α and NF-κB may constitute a loop to activate each other to promote the EMT program [133]. By inhibiting hypoxia-dependent HIF-1α and NF-κB activation, N-acetylcysteine (NAC) can suppress the expression of EMT regulators, such as Snail, Twist, and Slug in hypoxic areas of primary PC [134]. Moreover, triptolide can decrease HIF-1α-induced transcriptional signaling [122] and downregulate NF-κB activity [122, 135], which reverses hypoxia-induced EMT and stem-like features in PC [135].

Activated by hypoxia [136], Twist can act as a direct target of both HIF-1α and -2α to promote EMT in PC. In hypoxic PC cell lines, HIF-1α-mediated overexpression of Twist recruits EZH2 and Ring1B to trimethylate H3K27 and monoubiquitylate H2AK119, respectively. These histone modifications repress E-cadherin and p16 transcription by binding to the E-box (es) of their promoters, leading to hypoxia-induced EMT in PC [137]. HIF-2α promotes EMT of PC cell through the direct binding of Twist2 to the promoter of E-cadherin in -714 bp region site, rather than -295 bp promoter region site [138]. In contrast to Twist2, Twist1 has no binding capacity to neither of the above transcription regions of E-cadherin in PC. Which subtype of Twists binds to E-cadherin and contributes to EMT process appears to depend on different tumor types, and Twist2 is for PC [138]. In addition to Twist, other transcription factors also play a key role in EMT by negatively regulating adherens and tight junction proteins such as E-cadherin, claudins, occluding, and marvelD3. Snail can directly bind to E-boxes in the promoters of claudin/occludin genes and repress their expression, resulting in loss of epithelial cell polarity [139]. Activation of the HIF-1α-Snail regulatory axis induces EMT in hypoxic PC cells [128]. The high expression of protein kinase Cα (PKCα) downregulates claudin-1 and occludin via Snail- and MAPK/ERK dependent pathways, leading to EMT in PANC-1 cells treated with hypoxia or TGF-β1 treatment [140]. Another tight junction protein MarvelD3 is transcriptionally downregulated in Snail-induced EMT in PC cells as a result of hypoxia, TGF-β treatment, or knockdown of forkhead box transcription factor A2 (FOXA2), along with a decrease in cell polarity [141].

Intratumoral hypoxia is associated with the invasion and metastasis of PC [142]. The exceptionally high metastatic PDAC subpopulation is enriched for hypoxia-induced genes [143]. HIF-1α may be a dominant factor driving the metastatic progression of PC [144]. HIF-1-active cells constitute a large proportion of invasive and metastatic PC cells, whose eradication would compromise malignant progression in advanced PC [142]. The YEATS domain containing 2 (YEATS2) protein is a selective histone crotonylation reader that regulates the activity of histone deacetylases. HIF-1α increases YEATS2 expression via binding to its hypoxia response element (HRE) to promote the proliferation and migration of PC cells under hypoxia [145]. Moreover, HIF-1α mediates transcriptional activation of urokinase-type plasminogen activator receptor (uPAR) and uPAR-dependent angioinvasion, which is critical for hypoxia-induced metastasis [146]. LIM and SH3 protein 1 (LASP-1) is a direct target gene for HIF-1α, associated with actin assembly dynamics in cancer cells. HIF-1α stimulates LASP1 overexpression and promotes migration and invasion of PDAC cells in vitro and metastasis in xenograft mouse models [147]. Quiescin sulfhydryl oxidase 1 (QSOX1) is upregulated in human PC cell lines treated with hypoxia. HIF-1α induces QSOX1 expression by directly binding to two HREs of its gene, and subsequently, matrix metalloproteinase (MMP)-2 and MMP-9 are activated to promote hypoxia-induced PC cell invasion [148]. Hypoxia stimulates HIF-3α expression to a greater extent than HIF-1α and HIF-2α in multiple PC cell lines. HIF-3α promotes PC cell invasion and metastasis via transcriptional modulation of RhoC-ROCK1 signaling pathway which mediates the function of HIF-3α in controlling the cytoskeletal structure and stabilizing stress fibers [149]. There are several critical molecules involved in aggressive process of PDAC as well. Hypoxia-related expression of the enzyme lysyl oxidase (LOX) is increased in metastatic tumors from KPC mice and is required for mutant p53-driven invasion. LOX inhibition prevents both migration and invasion of KPC cells [150]. Ion channels are emerging factors in modulating cell migration. Under hypoxic microenvironment, the voltage dependent potassium channel K(V) 11.1 promotes PDAC cell migration through a complex regulation of f-actin assembly in stress fibers as well as formation and dynamics in filopodia [151]. Intratumoral hypoxia induces the expression of transcription factor Blimp1, which functions as a molecular switch that transiently promotes pro-metastatic gene expression and inhibits cell division in PDAC [143].

Interestingly, HIF-1α could even act as a tumor suppressor to prevent invasion and metastasis of PC cells. Deficiency of HIF-1α in PC cells enhances the expression of protein phosphatase 1 regulatory inhibitor subunit 1B (PPP1R1B), leading to phosphorylation of E3 ubiquitin-protein ligase (MDM2) at Ser166 and subsequent degradation of the p53 tumor suppressor protein. Loss of residual p53 significantly increases the metastatic potential of PC cells [152]. Moreover, genetic deletion of pancreas-specific HIF-1α drastically accelerates Kras^G12D^-driven PDAC neoplasia by enhancing B lymphocyte infiltration, indicating a protective role of HIF-1α in PC initiation [153]. Clinically, it was also demonstrated that weak HIF-1α expression correlates with poor prognosis in resectable PC and is an independent prognostic factor for PDAC-specific deaths [154]. From this perspective, the roles of HIF-1α in PC seem to be context dependent [152].

Activation of the Hh signaling pathway could mediate EMT in PC, concomitant with downregulated E-cadherin expression and upregulated vimentin expression [155, 156]. Hypoxia triggers smoothened (SMO) without affecting sonic hedgehog homolog (SHH) or patched1 (PTCH1) expression. SMO subsequently promotes GLI1 phosphorylation and permits nuclear accumulation of GLI1. GLI1 is responsible for transcriptional activity of target genes such as E-cadherin and vimentin. Additionally, some factors induced by hypoxia might bypass SMO to activate GLI1 directly, including TGF-β, KRAS or receptor tyrosine kinase (RTK). This SHH-independent process is described as non-canonical Hh signaling pathway and is required for a switch in hypoxia-induced EMT and invasion in PC cells [157]. Curcumin can suppress hypoxia-induced EMT by inhibiting the Hh signaling pathway in PC [158]. Through the Hh signaling pathway, hypoxia-induced ROS production elevates expression of metastatic-related factors, urokinase-type plasminogen activator (uPA) and MMP2, as well as invasive and migratory ability of PC cells [159]. The inhibitory effects of protein-bound polysaccharide (PSK) on proliferation and invasiveness in PDAC cells under hypoxia are attributed to the suppression of Hh signaling and HIF-1α pathways [160]. GLI1 is significantly increased by reoxygenation. GLI1 knockdown or SHH and SMO inhibition abrogates reoxygenation-induced increases in the invasiveness and metastasis of PDAC [161]. In addition to Hh signaling pathway, hypoxia-induced nuclear translocation of β-catenin also plays a role in initiating EMT [162]. In Bxpc-3 cells, β-catenin/EMT signaling mediated by hypoxia is blocked by forced expression of dickkopf-related protein 3 (DKK3) which is expressed at significantly lower levels in PC tissues than normal tissues due to its frequent methylation [163]. WNT7A, an important ligand of Wnt/β-catenin signaling pathways, presents significant high expression in PC tissue and is markedly upregulated in hypoxia culture condition. It might increase the migration capacity of cancer cells by enhancing EMT in PDAC [164].

Existing evidence shows that the aberrant expression of noncoding RNAs participates in hypoxia-induced aggressive and metastatic phenotypes of PC. Long non-coding RNA (lncRNA) NORAD is highly expressed in PDAC and upregulates during hypoxia. NORAD promotes EMT and metastasis under hypoxia by upregulating the small GTP binding protein RhoA in vitro and in vivo, which is attributed to the function of NORAD as a ceRNA to compete for miR-125a-3p [165]. In hypoxia-cultured CFPAC-1 and BxPC-3 cells, expression of miR-301a is increased by HIF-2α. Overexpression of miR-301a facilitates the hypoxia-induced EMT in PDAC cells by targeting downstream TP63 directly [166]. MiR-210 activates the NF-κB signaling pathway by inhibiting HOXA9 expression and mediates the occurrence of HIF-1α-induced EMT in hypoxic PC cells [132]. FEZF1‐AS1, a recently described oncogenic lncRNA, enhances PC cell proliferation and invasion ability by sponging the inhibitory effect of miR‐142 on HIF‐1α under hypoxia condition [167]. Increased expression of miR-224 suppresses thioredoxin-interacting protein (TXNIP) expression by targeting its 3′-UTR, leading to the nuclear translocation of HIF-1α and PDAC cell proliferation and migration [168]. In a hypoxia microenvironment, PC cell-derived exosomal miR-301a-3p polarizes M2 macrophages by activating PTEN/PI3K signaling pathway, which enhances metastatic potential of PC cells in vitro and in vivo [169]. Reintroduction of miR‑125a promotes mitochondrial fission in PANC‑1 cells through inhibition of Mfn2 transcription and expression. Extensive mitochondrial fission impairs the cellular migration capacity via induction of F‑actin depolymerization into G‑actin [170]. Table 1 shows the roles and underlying regulatory mechanisms of hypoxia-related noncoding RNAs on progression of PC [53, 104, 132, 165–187].

**Table 1:** Hypoxia-related noncoding RNAs in pancreatic cancer

ncRNAs	Expression in PC	Mechanisms	Main functions	References
*lncRNA*				
NORAD	Up	NORAD—miR-125a-3p—RhoA	Promotes EMT and metastasis	[165]
FEZF1-AS1	Up	FEZF1-AS1—miR-142—HIF-1α	Promotes cell proliferation and invasion ability	[167]
MTA2TR	Up	MTA2TR—ATF3—MTA2—HIF-1α loop	Promotes cell proliferation and invasion	[174]
BX111	Up	HIF-1α—YB1—ZEB1—E-cadherin, MMP2	Promotes growth and metastasis	[175]
NUTF2P3-001	Up	NUTF2P3-001—miR-3923—KRAS	Promotes the proliferation and invasion	[176]
CF129	Down	HIF-1α—CF129—p53—FOXC2 loop	Inhibits cell proliferation, invasion, and metastasis	[177]
*miRNA*				
circ_0000977	Up	circ_0000977—miR-153—HIF-1α, ADAM10	Promotes immune escape	[178]
miR-191	Up	HIF-1α—miR-191	Promotes tumorigenesis	[179]
miR-21	Up	miR-21—HIF-1α—VEGF, MMP2, MMP9	Promotes cell proliferation, migration, and invasion	[180]
		HIF-1α—miR-21	Avoids apoptosis	[181]
miR-210	Up	miR-210—HOXA9—NF-κB	Promotes EMT	[132]
miR-212	Up	HIF-1α—miR-212	Promotes progression	[182]
miR-224	Up	miR-224—TXNIP—HIF-1α	Promotes cell proliferation and migration	[168]
miR-301a	Up	HIF-2α—miR-301a—TP63	Promotes EMT	[166]
		miR-301a—TAp63		[173]
miR-301a-3p	Up	miR-301a-3p—PTEN/PI3K—M2 macrophages polarization	Promotes metastasis	[169]
miR-646	Up	HIF-1α—miR-646—MIIP—HDAC6 loop	Promotes proliferation and invasion	[183]
miR-124	Down	miR-124—MCT1—HIF-1α—LDHA	Increases intracellular pH, inhibits glycolytic activity and tumor growth	[53]
miR-125a	Down	miR-125a—Mfn2—mitochondrial fission	Impairs cell migration capacity	[170]
miR-138-5p	Down	miR-138-5p—SIRT1—FOXO1—Rab7	Inhibits autophagy and tumor growth	[104]
miR-142	Down	miR-142—HIF-1α—vimentin, VEGF-C, E-cadherin	Inhibits proliferation, EMT, and invasion	[184]
miR-150	Down	miRNA-150—CXCR4	Inhibits migration and invasion	[185]
miR-519	Down	miR-519—PD-L1	Inhibits cell invasiveness, and induces apoptosis	[186]
miR-548an	Down	HIF-1α—HDAC1—miR-548an—vimentin	Inhibits the proliferation and invasion	[187]

ADAM10, a disintegrin and metalloproteinase domain-containing protein 10; ATF3, activating transcription factor 3; FOXC2, forkhead box protein C2; FOXO1, forkhead box protein O1; HDAC, histone deacetylase; HOXA9, homeobox A9; LDHA, lactate dehydrogenase A; MCT1, monocarboxylate transporter 1; Mfn2, mitofusin 2; MIIP, migration and invasion inhibitory protein; MTA2, metastasis associated protein 2; ncRNAs, noncoding RNAs; RhoA, ras homolog family member A; SIRT1, sirtuin 1; TAp63, transactivation domain of tumor protein p63; TXNIP, thioredoxin-interacting protein; YB1, y box binding protein 1; ZEB1, zinc finger E-box-binding homeobox 1

The CSC characteristics of subpopulations of PDAC cells correlate with their aggressive and metastatic phenotypes. It was proposed that only PC cells with stemness properties acquire pronounced migratory potential with hypoxia-induced EMT [188]. Indeed, the dissemination and re-establishment of CSCs from the primary tumor to a secondary site are required for sustained metastatic growth [123]. Mechanistically, CD133 enhances N-cadherin expression via the Src/Slug signaling axis to facilitate EMT in PC [189]. Moreover, CD133 initiates the expression and transcriptional activity of HIF-1α, which encourages PC migratory and metastatic ability by inducing EMT gene transcription or autophagy under hypoxia [116, 123]. Furthermore, hypoxia induces a positive feedback loop of constitutive activation between nestin expression and the TGF-β1/Smad4 pathway [117]. As one of the pancreatic CSC markers, nestin is necessary to drive EMT and to promote cell migration and invasion in PDAC [114].

Hypoxic microenvironment remodeling by stromal cells also contributes to the aggressive and metastatic behavior of PC. Hypoxia stimulates HGF secretion from fibroblast MRC5 cells, which subsequently increases c-Met phosphorylation and MMP activity in PC PK8 cells. This hypoxic cancer-stromal crosstalk might be mediated by HIF-1α and enhances the invasive features of PC [190]. Moreover, hypoxia-induced procollagen-lysine, 2-oxoglutarate 5-dioxygenase 2 (PLOD2) expression in PSCs regulates the parallel patterning of ECM fiber architecture, which creates a permissive microenvironment for directional migration of PC cells [191]. Furthermore, increased secretion of IL-6 from hypoxic PSCs accelerates PC cell invasion, EMT, and metastasis through activation of ERK/NF‑κB axis, which can be counterbalanced by curcumin [192]. Depletion of CAFs suppresses immune surveillance by increasing the proportion of CD4^+^Foxp3^+^ Tregs and induces hypoxia, EMT, and CSC formation, leading to invasive and undifferentiated state of PDAC [193]. In contrast, it was also reported that arginase II expressed in CAFs after exposure to hypoxia mediates immune suppression in PC by reducing the level of arginine which is essential for T cell activity and survival [194]. Though the roles of CAFs in immune response under PC hypoxic microenvironment remain controversial, it is obvious that microenvironmental remodeling is a critical factor for fostering the metastatic ability of PDAC [143]. Figure 3 depicts the effects of microenvironmental remodeling on PC progression under hypoxic conditions.

### Pathological angiogenesis under hypoxia

HIFs are essential to hypoxia-induced angiogenesis in PC through the transcriptional activation of various proangiogenic factors, such as VEGF. Human clathrin heavy chain (CHC) contributes to the stability and nuclear translocation of HIF-1α, thereby promoting VEGF expression and angiogenesis in PC [195]. PHDs are the oxygen sensors regulating HIFs. Both PHD2 and PHD3 are hypoxia-inducible, and they can inhibit tumor growth through abrogation of HIF-1-dependent angiogenesis [196, 197]. NF-κB is activated to assist HIF-1α in enhancing the expression of angiogenic factors in the presence of hypoxia in PC [198]. Under hypoxic conditions, PKM2 and p65 are translocated to the nucleus, where PKM2 interacts with NF-κB/p65 to activate the transcription of HIF-1α and its target gene VEGF-A. As a result, increased secretion of VEGF causes a boost in blood vessel formation of hypoxic PC [199]. Moreover, overexpression of NF-κB increases transcription of heat shock protein 90 (Hsp90) and its client protein HIF-1α. Treatment with curcumin analogs inhibits transcription of HSP90, NF-κB, and HIF-1α, and thus displays antiangiogenic effects [200]. P-Stat3 is also a hypoxia-responsive nuclear transcription factor and can synergistically work with HIF-1α to regulate angiogenesis under hypoxia [201]. Hypoxia recruits the formation of the HIF-1α/p300/p-Stat3 complex, which facilitates VEGF production and angiogenesis. HIF-1α activation and Stat3 expression can be blocked by triptolidenol-1 via inhibition of the upstream PI3K/Akt/mTOR pathway [202]. Moreover, hypoxia-induced VEGF expression in PANC-1 cells is mediated by the ERK/AKT-HIF-1α-VEGF and Stat3-HIF-1α-VEGF signaling pathways, which can be targeted by α-solanine [201]. The Ape-1/Ref-1 redox domain function is required for the maintenance of pancreatic cancer-associated endothelial cells and endothelial progenitor cells, which contributes to tumor angiogenesis. Inhibition of the Ape-1/Ref-1 redox domain decreases the DNA binding activity of HIF-1α and reduces secreted and intracellular VEGF in PC cells [203]. MUC1 is hypoxia responsive in cells derived from metastatic PC and facilitates translocation of HIF-1α to the nucleus [204]. Furthermore, MUC1 overexpression activates the expression of multiple hypoxia-driven proangiogenic factors, including connective tissue growth factor (CTGF), VEGF-A, and platelet-derived growth factor-B (PDGFB), leading to endothelial cell tube formation in hypoxia-stressed PC cells [205]. Thus, MUC1 might promote pathological angiogenesis in hypoxic PC by stabilizing HIF-1α.

Although HIF-1α plays an essential role in the expression of proangiogenic cytokines, hypoxic PC cells can initiate an alternate pathway of angiogenesis in the absence of HIF-1α. Knockdown of HIF-1α stimulates the accumulation of glycogen due to the lack of HIF-1α-dependent glycogenolytic enzymes. Intracellular glycogen accumulation further drives the formation of immune-attractant inflammatory cytokines. Subsequently, conventional dendritic cells are recruited and release proangiogenic factors to sustain tumor vascularization [206]. Additionally, activation of protease-activated receptor-2 (PAR-2) augments MEK-ERK-mediated VEGF-A expression to promote angiogenesis in PC, independent of HIF-1α or HIF-2α [207]. Therefore, agents targeting HIFs only may be not enough to effectively prevent hypoxia-induced PC angiogenesis.

### Hypoxia-induced resistance to therapy

Apart from surgical resection, chemotherapy and radiotherapy are considered as the primary treatment approaches for PC patients at the present. It is generally recognized that chronic hypoxia is strongly associated with resistance to cytotoxic chemotherapy and radiotherapy of PC through various mechanisms. Gemcitabine is a standard first-line chemotherapeutic agent for PC patients as a single-agent therapy [208]. Gemcitabine promotes the degradation of mono-ADP ribosylated poly (ADP-ribose) polymerase-1 (PARP-1) through the autophagy pathway to induce DNA damage in PC cells. However, hypoxia reduces autophagic activity and thus suppresses gemcitabine-induced PARP-1 degradation, leading to gemcitabine resistance [209]. Gemcitabine treatment promotes PC cell stemness and resultant chemoresistance through activation of the AKT/Notch1 signaling cascade. This acquired gemcitabine resistance is synergistically aggravated by the hypoxic niche [6]. Clinical research has shown that high expression of L-type amino acid transporter 1 (LAT1) is closely correlated with hypoxia-induced genes and chemoresistance in PDAC patients [210]. The mRNA-binding protein HuR stabilizes the proto-oncogene PIM1 mRNA transcript and promotes hypoxia-induced PIM1-dependent chemoresistance in PDAC cells [211]. Hypoxia enhances the resistance of PC cells to gemcitabine-induced apoptosis mainly through the activation of PI3K/Akt/NF-κB signaling and partially through the MEK/ERK pathways [212]. Selective inhibition of G protein-coupled receptor 55 (GPR55) attenuates the MEK/ERK and PI3K-Akt pathways to diminish the expression and function of multidrug resistance (MDR) proteins such as P-glycoprotein (Pgp) and ATP-binding cassette subfamily G member 2 (ABCG_2_) in PANC-1 cells, resulting reduced resistance to doxorubicin and gemcitabine [213]. In Capan-2 cells, phosphorylated ERK1/2 upregulates ABCG_2_ expression through the binding of HIF-1α to its promoter and thus mediates drug resistance under hypoxic conditions [214].

HIFs play pivotal roles in hypoxia-induced therapy resistance of PC. High HIF-1α expression in PDAC cells markedly induces neomicrovascularity and reduces sensitivity to gemcitabine under hypoxia [215]. Inhibition of HIF-1α enhances chemosensitivity to gemcitabine in PC cells through suppression of autophagic flux [216]. IL-37/Stat3/HIF-1α constitutes a negative regulatory loop to decrease IL-37 expression in PDAC cells and drives gemcitabine resistance [217]. As a member of the P63 family, TAp63 reverses hypoxia-induced gemcitabine resistance via degradation of HIF-1α. MiR-301a inhibits its downstream target TAp63 and therefore promotes gemcitabine resistance [173]. HIF-1α is stabilized by MUC1 in gemcitabine-resistant PC cells, leading to glycolytic flux and de novo pyrimidine biosynthesis. Targeting HIF-1α or HIF-1α-mediated metabolic pathways improves the efficacy of gemcitabine [33]. HIF-2α induces expression of the SLC1A5 variant under hypoxia, which promotes mitochondrial glutamine metabolism and confers gemcitabine resistance to PC cells [60]. Irradiation upregulates HIF-1α expression in PDAC cells and conversely mediates radioresistance. Inhibition of HSP90 overcomes HIF-1α-induced radioresistance and potentiates the antitumor effects of chemoradiotherapy [218]. PHD3 is downregulated in PC cells, but its overexpression can exacerbate irradiation-induced apoptosis and improve radiotherapy efficacy by promoting the degradation of HIF-1α, especially under hypoxia [219].

Gemcitabine chemoresistance can be mediated by modulation of redox homeostasis. ROS induction upon gemcitabine treatment results in the nuclear accumulation of NF-κB and HIF-1α through activation of ERK1/2 and Akt. Enhanced binding of NF-κB and HIF-1α to the CXCR4 promoter upregulates the expression of CXCR4, which facilitates the migration and invasion of gemcitabine-treated PC cells and mediates acquired gemcitabine resistance [220]. Consistently, the ROS scavenger NAC sensitizes PC to gemcitabine by inactivating the NF-κB pathway [221]. In contrast, elevated ROS levels under hypoxia have also been demonstrated to improve gemcitabine chemotherapeutic efficacy. For example, silencing of enolase 1 (ENO1), a multifunctional glycolytic enzyme, sensitizes PC cells to hypoxia-induced chemoresistance by increasing intracellular ROS [222].

Notably, some targeted inhibitors in clinical trials may decrease the chemosensitivity of hypoxic PDAC cells to some chemotherapy drugs. For example, the SMO inhibitor cyclopamine significantly increases chemoresistance to 5-FU and gemcitabine under hypoxia [223]. Therefore, interference between agents administrated in vivo should be fully taken into account when combination therapies are used.

## Hypoxia-based therapeutic strategies for PC

### Strategies to ameliorate blood vessel perfusion and lessen tissue hypoxia

PC is characterized by structural and functional abnormalities in blood vessels that impair vascular perfusion and increase hypoxia-associated malignant phenotypes, severely affecting antineoplastic drug delivery and radiotherapy effectiveness [224]. Therefore, vascular normalizing strategies aimed at ameliorating cancer perfusion and lessening tissue hypoxia have been developed to hamper PC progression. The semaphorin-3A (SEMA3A) point mutant isoform displays stronger activity on the inhibition of endothelial cells (ECs) in PC models due to its high affinity to bind Plexin A4 (PLXNA4) compared with its wild-type counterpart. Owing to this feature, the mutated SEMA3A protein isoform successfully normalizes the vasculature, curbs tumor growth and metastatic progression, and effectively improves the sensitivity to chemotherapy [225]. The nucleolin antagonist N6L markedly increases pericyte coverage and vessel perfusion and inhibits tumor hypoxia in PDAC mice, in parallel to decreased angiopoietin-2 (Ang-2) secretion and expression in ECs. As a consequence of tumor vascular normalization, pretreatment with N6L efficiently improves the delivery and efficacy of chemotherapeutic drugs. Ang-2 in the plasma may be a suitable response biomarker for N6L treatment in PC [226]. Myo-inositol trispyrophosphate (ITPP) treatment restores PC normoxia in the rapid early and sustainable late stage by normalizing vessel structure and decreasing desmoplasia, resulting in elevated immune cell influx, enhanced chemotherapy susceptibility and synergistic life prolongation [227]. Intravenous administration of vascular proangiogenic cells derived from cultured bone marrow mononuclear cells in vitro into pancreatic tumor-bearing mice triggers a functional normalization of the tumor vasculature and a marked reduction in hallmarks involved in drug resistance and cancer cell stemness [228]. Unlike the inhibitory effects of vascular normalization strategies on angiogenic factors, the protein phosphatase 2A (PP2A) inhibitor LB-100 increases microvessel density and blood perfusion via HIF-1α-VEGF-mediated angiogenesis to enhance the cytotoxicity of doxorubicin in PC models [229].

Reducing pressure from solid tissue components, namely solid stress, is considered another effective strategy to ameliorate blood vessel perfusion. Hyaluronan (HA) is implicated as the primary tumor interstitial matrix molecule to compress PDAC blood vessels and limit vascular perfusion due to its swelling behavior [230]. The accumulation of HA in tumors is accompanied by vascular collapse, hypoxia, and drug resistance [231]. Enzymatic degradation of HA mediated by PEGylated PH20 hyaluronidase (PEGPH20) leads to vascular expansion, reduced hypoxia, increased extracellular pH, and depleted stores of VEGF-A165 in HA-accumulating tumors, including PC [231]. Collagen collaborates with HA to compress PC blood vessels. The angiotensin inhibitor losartan not only reduces stromal collagen and HA production but also decreases stromal fibrosis signaling and consequently increases vascular perfusion and potentiates chemotherapy [232]. LOX inhibition synergizes with gemcitabine to kill tumors through stromal alterations, including decreased fibrillar collagen, increased vasculature, and elevated infiltration of macrophages and neutrophils into PC [150].

Metronomic gemcitabine treatment is also a preferable approach to improve perfusion and reduce hypoxia in human PDAC. Metronomic gemcitabine-treated PC exhibits consistent antitumor efficacy but better tissue perfusion and less toxicities than their maximum tolerated dose (MTD)-driven regimens, concomitant with a prominent reduction in hypoxia, CAFs, and multiple proangiogenic factors [233]. The therapeutic efficacy of metronomic gemcitabine is markedly superior to that of the dedicated antiangiogenic agent DC101 in PC [234]. In terms of their activity modes, compared with DC10-treated tumors, metronomic gemcitabine-treated tumors have a higher and more functional vessel density, which decreases hypoxia, and they are better perfused with blood flow that is uniformly distributed in the tumor rather than confined to the periphery [234].

### Strategies to decrease vascular perfusion and target the hypoxic tumor microenvironment

In contrast to vascular normalizing strategies, starvation therapy engineered by an extravascular gelation shrinkage-derived internal stress strategy reduces vessel density, occludes blood supply and tumor migration passages, and induces hypoxia and apoptosis, thereby suppressing PANC-1 PC growth, metastasis, and recurrence [235]. Apart from squeezing and narrowing blood vessels by increasing extravascular pressure, tumor angiogenesis is targeted to realize starvation therapy as well. The tyrosine kinase inhibitor BIBF 1120 (nintedanib) displays potent antiangiogenic effects evidenced by decreases in microvessel density, pericyte coverage, vessel permeability, and perfusion in preclinical models of PC. Despite being accompanied by increased hypoxia, EMT is not induced in BIBF 1120-treated tumors [236]. As an angiogenesis and heparanase inhibitor, PG545 reduces microvessel density, disrupts vascular function, and elevates intratumoral hypoxia but avoids hypoxia-derived collagen deposition and PDAC progression [237]. Cyclooxygenase-2 (COX-2) inhibition can potentiate the efficacy of anti-VEGF therapy by reversing hypoxia-induced EMT and promoting an immune landscape that increases tumor-associated CD8^+^ T cells while reducing FoxP3^+^ T cells and FasL expression on the tumor endothelium [238]. The antitumoral activity of propofol is associated with its antagonization of neovascularization by inhibiting the activation and expression of ADAM8 in response to hypoxia in PC [239]. The hypoxic cell radiosensitizer TX-1877 not only enhances the hypoxic radioresponse in PC but also shows the antitumor activity through suppression of angiogenesis and metastasis [240]. Compared to the potential protumor risks brought by increased hypoxia during antiangiogenic therapy, Fuco-MnO_2_-NPs dually target tumor hypoxia and angiogenesis, which reverses radioresistance [241].

Hypoxia-activated prodrugs (HAPs) are relatively inert in tissues with physiological pO_2_ levels but are specifically activated under hypoxic conditions to release cytotoxic or cytostatic effectors, therefore improving antitumor efficacy while lowering side effects from nonspecific toxicities [242]. The hypoxia-activated cytotoxin AQ4N can readily penetrate BxPC-3 tumors and converse into the potent antineoplastic metabolite AQ4, which rapidly accumulates in tumor tissues. Tumor growth, progression, and survival are significantly delayed in a manner comparable to gemcitabine after single-agent administration of AQ4N in multiple preclinical models of PC [243]. Incorporation of the hypoxia-targeted agent TH-302 into ionizing radiation (IR) therapy is particularly effective in treating more rapidly growing and hypoxic patient-derived pancreatic xenografts (PDXs) [244]. Hydralazine enhances the efficacy of TH-302 in PC by acutely reducing tumor perfusion and exacerbating hypoxia within the tumor microenvironment, although the effect size is small [245]. Nonpharmacological methods, such as supplying exogenous pyruvate, also improve TH-302 activity by transiently increasing tumor hypoxia. Combination therapy of pyruvate plus TH-302 significantly decreases tumor growth and increases survival in multiple PC models [246]. To overcome the barrier that the deep-located hypoxic regions are usually less accessible to intravenously injected drug carriers or drugs, hypoxia-responsive lipid nanoparticles (LNs) that encapsulate gemcitabine in the aqueous core have been designed. LNs penetrate deeper and release gemcitabine to the hypoxic cores by reducing and destabilizing the lipid membrane under a low pO_2_, resulting in decreased viability and enhanced cytotoxicity in the cultured PC cell spheroids [247].

### Strategies targeting HIF signaling pathways

HIFs regulate the transcription of many genes in response to hypoxia and facilitate malignant phenotypes of PC, so targeting HIFs and related signaling pathways may assist in PC therapy. Hypoxic cytotoxin TX-2098 exerts antitumor efficacy and prolongs survival in xenograft models of PC by inhibiting the expression of HIF-1α and HIF-1α-associated molecules such as VEGF, glucose transporter 1, and aldolase A [248]. Compound 9o, one of the benzofuran derivatives, dramatically suppresses PDAC growth in nude mice via the HIF-1α/VEGF pathway under hypoxic conditions [249]. A novel in vivo CRISPR/Cas9 delivery system constructed by R8-dGR-modified cationic liposomes penetrates deeply into PC spheroids and enhances the antiproliferation and antimetastatic effect of paclitaxel in the BxPC-3 PC model through downregulation of HIF-1α and its downstream molecules VEGF and MMP9, without severe toxicity induced in vivo [250]. Codelivery of gemcitabine and HIF-1α siRNA via lipid ε-polylysine copolymers (LENPs) leads to better stability and prolonged circulating time in the bloodstream than delivery of the free components and prevents innate immune activation by siRNA. This codelivery system effectively suppresses HIF-1α expression and consequently exhibits significant synergistic antitumor and antimetastatic effects in vivo [251]. Combined treatment with gemcitabine and the selective HIF-1α inhibitor PX-478 significantly inhibits PC growth by eliciting immunogenic cell death, because IFN-γ secretion by cytotoxic T lymphocytes and dendritic cell maturation/phagocytosis are induced only when the two agents are combined [252]. Moreover, PX-478 is also an enhancer of PC radiosensitivity, contributing to acute tumor microvessel decompression and increased tumor blood flow through inhibition of tumoral and stromal HIF-1 proangiogenic signaling [253]. However, it has also been proposed that HIF-1α deletion is likely to cause pleiotropic effects, for example, affecting the release of tumor suppressive pathways and accelerating PC growth [153]. Therefore, targeting specific downstream effectors rather than HIF-1α itself may be a more specific and less toxic therapeutic strategy [41]. Inhibition of LDHA by FX11 decreases ATP levels, induces significant oxidative stress, increases cell death, and consequently suppresses the progression of sizable PC xenografts [254]. LDHA inhibitors also display synergistic cytotoxic effects with gemcitabine in hypoxic PDAC cells by modulating gemcitabine metabolism and restoring the synthesis of phosphorylated metabolites [255]. Knockdown or pharmacologic inhibition of CA9 in PDAC cells reduces gemcitabine-induced glycolysis and overcomes hypoxia-induced gemcitabine resistance. Combined administration of the CA9 inhibitor SLC-0111 and gemcitabine significantly slows tumor growth in multiple PC models [41].

### Application of dynamic therapies on hypoxic PC

Dynamic therapies are dependent on the production of ROS derived from O_2_ and/or reducible chemical species present in tumor cells or tissues. As essential energy sources of dynamic therapies, light and sound have laid solid foundations in photodynamic therapy (PDT) and sonodynamic therapy (SDT), separately. Since O_2_ is a key substrate for dynamic therapies, alternative strategies for supplying O_2_ to hypoxic tissue are necessary to treat PC with these techniques [256]. A CaO_2_-containing nanoparticle formulation harbors a pH-responsive polymer coat that enables the release of CaO_2_ particles to water, resulting in molecular oxygen generation at a low environmental pH. Subsequent significantly elevated tumor pO_2_ dramatically improves the efficacy of PDT treatment on PC growth [257]. A type of oxygen self-sufficient PDT platform constructed by zeolite-catalase-methylene blue (ZCM) nanocapsules has also been developed. Upon implantation into the tumor area of mice, the nanocapsule efficiently overcomes tumor hypoxia through sustained decomposition of endogenous H_2_O_2_ and in situ release of highly sustained O_2_ gas bubbles. As a consequence, the local PC cells are completely killed upon near-infrared laser irradiation without therapy-induced toxicity and recurrence [258]. The synthesized RuTe hollow nanorods (RuTeNRs) exhibit peroxidase-SOD-catalase-like nanozymatic activity but lack oxidase-like activity and thus accelerate the efficient and continuous generation of O_2_ and ROS, which are critical factors for RuTeNR-mediated hypoxic PC PDT under multiwavelength laser irradiation [259]. Compared to the relatively shallow penetration depth of light in PDT, ultrasound (US) triggers deeper penetration in SDT, and therefore, SDT is preferable over PDT for treating deep tumors [260]. Compared to the use of oxygen-loaded, lipid-stabilized microbubbles alone, their use in combination with a sensitizer drug enhances SDT in a hypoxic BxPc-3 PC model, with more singlet oxygen generated, a greater cytotoxic effect observed, and a significant reduction in tumor volume [256]. An oxygen-self-produced SDT nanoplatform involving a modified fluorocarbon chain-mediated oxygen delivery method relieves hypoxia and produces sufficient ROS to shrink hypoxic PANC-1 PC [261]. These oxygen delivery protocols hold great potential for reducing hypoxia-induced resistance to oxygen-depleting therapies, which include but are not limited to dynamic therapies, radiotherapy, and chemotherapy.

**Fig. 4:**
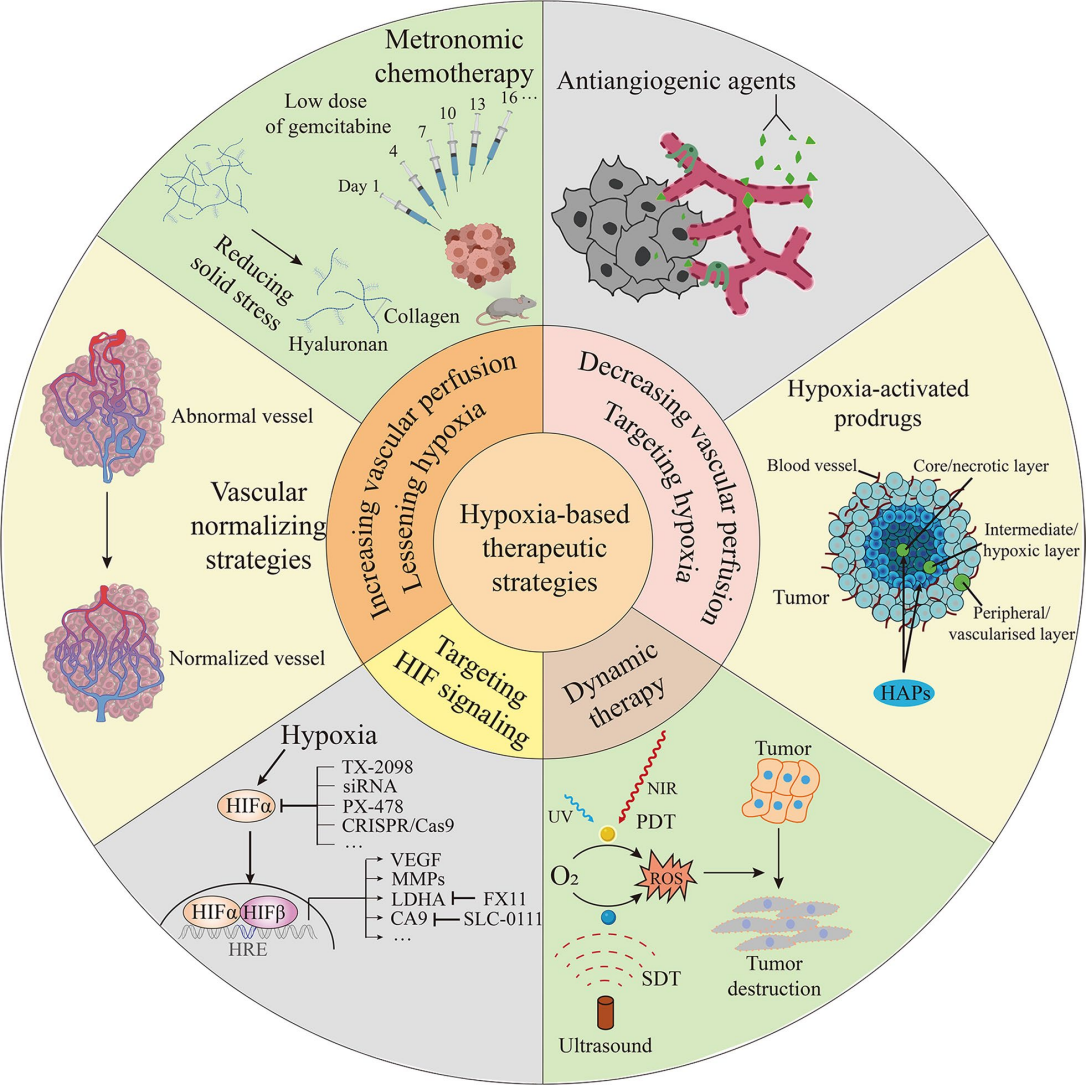
Summary of hypoxia-based therapeutic strategies for PC. Arrows indicate positive modulations or transitions, while blunt ends indicate negative modulations. Abbreviations: HAPs, hypoxia-activated prodrugs; NIR, near-infrared radiation; PDT, photodynamic therapy; SDT, sonodynamic therapy; UV, ultraviolet

Hypoxia-based therapeutic strategies are summarized in Fig. 4, some of which have been applied to clinical trials to evaluate their efficacy and safety in the treatment of PC (as seen in Table 2). To date, only a modest number of clinical trials have been performed in the initial stages. They focused primarily on the combined use of hypoxic agents with chemotherapy and/or radiotherapy. More attempts should be made to translational research on hypoxia-targeted strategies to identify novel treatment options for PC patients in the future.

**Table 2:** Hypoxia-targeted clinical trials in pancreatic cancer

Categories	Interventions	Pancreatic Cancer Types	Phases	Status	NCT Number
Vascular normalizing agent	*OXY111A* (*ITPP*)	Pancreatic Neoplasms	I, II	N/A	NCT02528526
	Chemoradiotherapy (CRT) (nab-paclitaxel, Capecitabine, Gemcitabine, high/low dose of radiation) ± *Nelfinavir*	Pancreatic Neoplasms (Locally Advanced Non-metastatic)	I, II	Recruiting	NCT02024009
	*Cediranib/Cediranib Maleate* + Olaparib	Metastatic Pancreatic Adenocarcinoma, Stage III Pancreatic Cancer AJCC v6 and v7, Stage IV Pancreatic Cancer AJCC v6 and v7, Unresectable Pancreatic Carcinoma	II	Recruiting	NCT02498613
Antiangiogenic agent	*Bevacizumab* (Anti-VEGF monoclonal antibody) + Capecitabine + Radiation	Locally Advanced Pancreatic Cancer	I	Completed	NCT00047710
	*Sorafenib* + Gemcitabine	Pancreatic Cancer, Adenocarcinoma of the Pancreas	I	Completed	NCT00375310
Hypoxia-activated prodrug	*TH-302* (*Evofosfamide*) + Gemcitabine/ Docetaxel/Pemetrexed	Pancreatic Cancer	I, II	Completed	NCT00743379
	Gemcitabine + *TH-302*/Placebo	Metastatic or Locally Advanced Unresectable Pancreatic Adenocarcinoma	III	Completed	NCT01746979
	*TH-302* + Sunitinib	Pancreatic Neuroendocrine Tumors	I	N/A	NCT01381822
	*TH-302* + Ipilimumab	Metastatic Pancreatic Cancer	I	N/A	NCT03098160
Inhibition of HIF-1α	*Digoxin* + FOLFIRINOX (5-Fluorouracil, Calcium Leucovorin, Irinotecan, Oxaliplatin)	Pancreas Cancer; Adenocarcinoma of the Pancreas	II	Not yet recruiting	NCT04141995
Photodynamic therapy	EF5 (a surrogate marker for hypoxia) + *Motexafin Lutetium* (photosensitizing drug)	Recurrent and Stage I to IV Pancreatic Cancer	N/A	Terminated	NCT00087191

The italics in the “Interventions” column represent drugs used for hypoxia-based therapeutic strategies. N/A, not available

## Conclusion

PC exhibits higher levels of hypoxia than most solid tumors [9], and the presence of intratumoral hypoxia is associated with an unfavorable prognosis in PC patients. As the master transcription factors that regulate adaptive responses to changes in tissue oxygenation, HIFs are highly involved in various hypoxia-induced malignant phenotypes of PC. These phenotypes have close correlations with each other, and their molecular mechanisms constitute a signaling network in hypoxic microenvironment of PC (as shown in Fig. 2). However, an increasing number of studies have shown that HIFs do not always act as tumor promoters to facilitate PC progression and targeting HIF-1α is likely to induce pleiotropic effects due to transcription of genes with various biological functions. In addition, hypoxic PC cells can initiate alternative pathways independent of HIFs to assist in PC development. Therefore, strategies to target hypoxic tumor microenvironment itself may be more effective and less toxic in treatments against PC than those HIF-targeted only. Notably, requirement for oxygen allows PC cells to grow in hypoxia but cannot support their growth in anoxia [262], and reoxygenation causes oxidative injuries to PC cells. Based on this theory, strategies to lessen tissue hypoxia or to further exacerbate and target hypoxia are exploited to innovative therapies for PC. Moreover, dynamic therapies provide oxygen delivery protocols to overcome resistance of PC to chemotherapy and radiotherapy, the current primary treatment options apart from surgical resection for PC patients. More translational research is urgently needed to implement hypoxia-targeted therapies in clinical practice.

## Data Availability

Not applicable.

## Abbreviations

3′-UTR: 3′-Untranslated region
α-KG: α-Ketoglutarate
α-KGDH: α-Ketoglutarate dehydrogenase
ABCG_2_: ATP-binding cassette subfamily G member 2
ACSS2: Short chain acyl-CoA synthetases 2
AMPK: AMP-activated protein kinase
Ang-2: Angiopoietin-2
APE1/Ref-1: AP endonuclease-1/redox effector factor 1
AURKA: Aurora kinase A
BCRP: Breast cancer resistance protein
CA9: Carbonic anhydrase IX
CAFs: Cancer-associated fibroblasts
cav-1: Caveolin-1
CHC: Clathrin heavy chain
COX-2: Cyclooxygenase-2
CSC: Cancer stem cell
CTGF: Connective tissue growth factor
CXCR4: C-X-C chemokine receptor type 4
DKK3: Dickkopf-related protein 3
dnHIF-1α: Dominant-negative HIF-1α
ECM: Extracellular matrix
EGLN2: Egl-9 family hypoxia inducible factor 2
EMT: Epithelial-mesenchymal transition
ENO1: Enolase 1
ERO1L: Endoplasmic reticulum oxidoreductase 1
ESA: Epithelial surface antigen
Fbln5: Fibulin-5
FOXA2: Forkhead box transcription factor A2
Fuco-MnO2-NPs: Fucoidan-coated manganese dioxide nanoparticles
GClc: Glutamate-cysteine ligase
GDH: Glutamate dehydrogenase
Gln: Glutamine
GLS: Glutaminase
Glu: Glutamine
GLUT1: Glucose transporter 1
GOT1: Cytoplasmic aspartate transaminase
GOT2: Mitochondrial aspartate transaminase
GPR55: G protein-coupled receptor 55
GPX4: Glutathione peroxidase 4
H/R: Hypoxia and reoxygenation
H_2_O_2_: Hydrogen peroxide
HAPs: Hypoxia-activated prodrugs
HGF: Hepatocyte growth factor
Hh: Hedgehog
HIF: Hypoxia-inducible factors
HK2: Hexokinase 2
HO-1: Heme oxygenase-1
HRE: Hypoxia response element
HSP90: Heat shock protein 90
IDH1: Isocitrate dehydrogenase 1
IGF1R: IGF-1 receptor
IL: Interleukin
IR: Insulin receptor
ITPP: Myo-inositol trispyrophosphate
Kras^G12D^-LOH: Loss of heterozygosity for Kras^G12D^
LASP-1: LIM and SH3 protein 1
LAT1: L-type amino acid transporter
1LDH: Lactate dehydrogenase
LENPs: Lipid ε-polylysine copolymers
LMP: Lysosomal membrane permeabilization
lncRNA: Long non-coding RNA
LNs: Lipid nanoparticles
LOX: Lysyl oxidase
LSD1: Lysine specific demethylase 1
MCT: Monocarboxylate transporter
MDH1: Malate dehydrogenase
MDM2: E3 ubiquitin-protein ligase
MDR: Multidrug resistance
ME1: Malic enzyme
mFOLFIRINOX: Modified FOLFIRINOX
MMP: Matrix metalloproteinase
MTD: Maximum tolerated dose
MUC: Mucin
NAC: N-acetylcysteine
NADPH: Nicotinamide adenine dinucleotide phosphate
NDRG1: N-myc downstream regulator gene 1
NFAT5: Nuclear factor of activated T cells 5
NQO1: NAD(P)H quinone oxidoreductase 1
OAA: Oxaloacetate
OPN: Osteopontin
p300: Acetyltransferase
P4HA1: Prolyl 4-hydroxylase subunit alpha 1
PAR-2: Protease-activated receptor-2
PARP-1: Poly (ADP-ribose) polymerase-1
P-AscH^−^: Pharmacological ascorbate
PC: Pancreatic cancer
PDAC: Pancreatic ductal adenocarcinoma
PDGFB: Platelet-derived growth factor-B
PDK1: Pyruvate dehydrogenase kinase-1
PDT: Photodynamic therapy
PDX: Patient-derived pancreatic xenografts
PEGPH20: PEGylated PH20 hyaluronidase
PGK-1: Phosphoglycerate kinase 1
Pgp: P-glycoprotein
PHD: Prolyl hydroxylase
PKCα: Protein kinase Cα
PKCδ: Protein kinase C-delta
PKM2: Pyruvate kinase M2
PLOD2: Procollagen-lysine, 2-oxoglutarate 5-dioxygenase 2
PLXNA4: Plexin A4
PO_2_: Partial oxygen pressure
PPP1R1B: Protein phosphatase 1 regulatory inhibitor subunit 1B
PSCs: Pancreatic stellate cells
PSK: Protein-bound polysaccharide
PTBP3: Polypyrimidine tract-binding protein 3
PTCH1: Patched1
QSOX1: Quiescin sulfhydryl oxidase 1
Rac1: Rac GTPase 1
REDD1: Regulated in DNA damage and development 1
RER1: Retention in endoplasmic reticulum 1
ROS: Reactive oxygen species
RTK: Receptor tyrosine kinase
RuTeNRs: RuTe hollow nanorods
SDF-1: Stromal cell-derived factor-1
SDT: Sonodynamic therapy
SEMA3A: Semaphorin-3A
SHH: Sonic hedgehog homolog
SIRT: Sirtuin
SMO: Smoothened
SOD: Superoxidase dismutase
SP: Side population
TCA: Tricarboxylic acid
TCF7L2/TCF4: Transcription factor 7-like2/transcription factor 4
TG2: Tissue transglutaminase
TXNIP: Thioredoxin-interacting protein
UHRF1: Ubiquitin like with plant homeodomain (PHD) and ring finger domains 1
uPA: Urokinase-type plasminogen activator
uPAR: Urokinase-type plasminogen activator receptor
US: Ultrasound
VEGF: Vascular endothelial growth factor
YEATS2: YEATS domain containing 2
ZCM: Zeolite-catalase-methylene blue

## References

[1] Siegel RL, Miller KD, Jemal A (2020). Cancer statistics, 2020. CA Cancer J Clin.

[2] Chen W, Zheng R, Baade PD, Zhang S, Zeng H, Bray F (2016). Cancer statistics in China, 2015. CA Cancer J Clin.

[3] Kleeff J, Reiser C, Hinz U, Bachmann J, Debus J, Jaeger D (2007). Surgery for recurrent pancreatic ductal adenocarcinoma. Ann Surg.

[4] Mizrahi JD, Surana R, Valle JW, Shroff RT (2020). Pancreatic cancer. Lancet.

[5] Leonhardt CS, Traub B, Hackert T, Klaiber U, Strobel O, Büchler MW (2020). Adjuvant and neoadjuvant chemotherapy in pancreatic ductal adenocarcinoma. J Pancreatol.

[6] Zhang Z, Han H, Rong Y, Zhu K, Zhu Z, Tang Z (2018). Hypoxia potentiates gemcitabine-induced stemness in pancreatic cancer cells through AKT/Notch1 signaling. J Exp Clin Cancer Res.

[7] Feng M, Xiong G, Cao Z, Yang G, Zheng S, Song X (2017). PD-1/PD-L1 and immunotherapy for pancreatic cancer. Cancer Lett.

[8] Mosquera C, Maglic D, Zervos EE (2016). Molecular targeted therapy for pancreatic adenocarcinoma: a review of completed and ongoing late phase clinical trials. Cancer Genet.

[9] Koong AC, Mehta VK, Le QT, Fisher GA, Terris DJ, Brown JM (2000). Pancreatic tumors show high levels of hypoxia. Int J Radiat Oncol Biol Phys.

[10] Hu Q, Qin Y, Ji S, Xu W, Liu W, Sun Q (2019). UHRF1 promotes aerobic glycolysis and proliferation via suppression of SIRT4 in pancreatic cancer. Cancer Lett.

[11] Erkan M, Hausmann S, Michalski CW, Fingerle AA, Dobritz M, Kleeff J (2012). The role of stroma in pancreatic cancer: diagnostic and therapeutic implications. Nat Rev Gastroenterol Hepatol.

[12] Qin Y, Zhu W, Xu W, Zhang B, Shi S, Ji S (2014). LSD1 sustains pancreatic cancer growth via maintaining HIF1α-dependent glycolytic process. Cancer Lett.

[13] Harris AL (2002). Hypoxia–a key regulatory factor in tumour growth. Nat Rev Cancer.

[14] Jiang BH, Rue E, Wang GL, Roe R, Semenza GL (1996). Dimerization, DNA binding, and transactivation properties of hypoxia-inducible factor 1. J Biol Chem.

[15] Hu CJ, Wang LY, Chodosh LA, Keith B, Simon MC (2003). Differential roles of hypoxia-inducible factor 1alpha (HIF-1alpha) and HIF-2alpha in hypoxic gene regulation. Mol Cell Biol.

[16] Jokilehto T, Jaakkola PM (2010). The role of HIF prolyl hydroxylases in tumour growth. J Cell Mol Med.

[17] Raz S, Sheban D, Gonen N, Stark M, Berman B, Assaraf YG (2014). Severe hypoxia induces complete antifolate resistance in carcinoma cells due to cell cycle arrest. Cell Death Dis.

[18] Parks SK, Mazure NM, Counillon L, Pouyssegur J (2013). Hypoxia promotes tumor cell survival in acidic conditions by preserving ATP levels. J Cell Physiol.

[19] Uchida T, Rossignol F, Matthay MA, Mounier R, Couette S, Clottes E (2004). Prolonged hypoxia differentially regulates hypoxia-inducible factor (HIF)-1alpha and HIF-2alpha expression in lung epithelial cells: implication of natural antisense HIF-1alpha. J Biol Chem.

[20] Bristow RG, Hill RP (2008). Hypoxia and metabolism. Hypoxia, DNA repair and genetic instability. Nat Rev Cancer..

[21] Chiche J, Brahimi-Horn MC, Pouysségur J (2010). Tumour hypoxia induces a metabolic shift causing acidosis: a common feature in cancer. J Cell Mol Med.

[22] DeBerardinis RJ, Mancuso A, Daikhin E, Nissim I, Yudkoff M, Wehrli S (2007). Beyond aerobic glycolysis: transformed cells can engage in glutamine metabolism that exceeds the requirement for protein and nucleotide synthesis. Proc Natl Acad Sci USA.

[23] Mikuriya K, Kuramitsu Y, Ryozawa S, Fujimoto M, Mori S, Oka M (2007). Expression of glycolytic enzymes is increased in pancreatic cancerous tissues as evidenced by proteomic profiling by two-dimensional electrophoresis and liquid chromatography-mass spectrometry/mass spectrometry. Int J Oncol.

[24] Guillaumond F, Leca J, Olivares O, Lavaut MN, Vidal N, Berthezene P (2013). Strengthened glycolysis under hypoxia supports tumor symbiosis and hexosamine biosynthesis in pancreatic adenocarcinoma. Proc Natl Acad Sci U S A.

[25] Hu M, Chen X, Ma L, Ma Y, Li Y, Song H (2019). AMPK Inhibition suppresses the malignant phenotype of pancreatic cancer cells in part by attenuating aerobic glycolysis. J Cancer.

[26] Jiang Y, He R, Jiang Y, Liu D, Tao L, Yang M (2019). Transcription factor NFAT5 contributes to the glycolytic phenotype rewiring and pancreatic cancer progression via transcription of PGK1. Cell Death Dis.

[27] Gunda V, Kumar S, Dasgupta A, Singh PK (2018). Hypoxia-induced metabolomic alterations in pancreatic cancer cells. Methods Mol Biol.

[28] Keith B, Simon MC (2007). Hypoxia-inducible factors, stem cells, and cancer. Cell.

[29] He G, Jiang Y, Zhang B, Wu G (2014). The effect of HIF-1α on glucose metabolism, growth and apoptosis of pancreatic cancerous cells. Asia Pac J Clin Nutr.

[30] Chen J, Zhao S, Nakada K, Kuge Y, Tamaki N, Okada F (2003). Dominant-negative hypoxia-inducible factor-1 alpha reduces tumorigenicity of pancreatic cancer cells through the suppression of glucose metabolism. Am J Pathol.

[31] Cao XP, Cao Y, Li WJ, Zhang HH, Zhu ZM (2019). P4HA1/HIF1alpha feedback loop drives the glycolytic and malignant phenotypes of pancreatic cancer. Biochem Biophys Res Commun.

[32] Chaika NV, Gebregiworgis T, Lewallen ME, Purohit V, Radhakrishnan P, Liu X (2012). MUC1 mucin stabilizes and activates hypoxia-inducible factor 1 alpha to regulate metabolism in pancreatic cancer. Proc Natl Acad Sci USA.

[33] Shukla SK, Purohit V, Mehla K, Gunda V, Chaika NV, Vernucci E (2017). MUC1 and HIF-1alpha signaling crosstalk induces anabolic glucose metabolism to impart gemcitabine resistance to pancreatic cancer. Cancer Cell.

[34] Shi S, Xu J, Zhang B, Ji S, Xu W, Liu J (2016). VEGF Promotes glycolysis in pancreatic cancer via HIF1α Up-regulation. Curr Mol Med.

[35] Logsdon DP, Shah F, Carta F, Supuran CT, Kamocka M, Jacobsen MH (2018). Blocking HIF signaling via novel inhibitors of CA9 and APE1/Ref-1 dramatically affects pancreatic cancer cell survival. Sci Rep.

[36] Zou G-M, Maitra A (2008). Small-molecule inhibitor of the AP endonuclease 1/REF-1 E3330 inhibits pancreatic cancer cell growth and migration. Mol Cancer Ther.

[37] Logsdon DP, Grimard M, Luo M, Shahda S, Jiang Y, Tong Y (2016). Regulation of HIF1α under Hypoxia by APE1/Ref-1 Impacts CA9 Expression: Dual Targeting in Patient-Derived 3D Pancreatic Cancer Models. Mol Cancer Ther.

[38] Raphael BJ, Hruban RH, Aguirre AJ, Moffitt RA, Yeh JJ, Stewart C, Gabriel SB (2017). Integrated genomic characterization of pancreatic ductal adenocarcinoma. Cancer cell.

[39] Ying H, Kimmelman AC, Lyssiotis CA, Hua S, Chu GC, Fletcher-Sananikone E (2012). Oncogenic Kras maintains pancreatic tumors through regulation of anabolic glucose metabolism. Cell.

[40] Zhang X, Ma N, Yao W, Li S, Ren Z (2019). RAD51 is a potential marker for prognosis and regulates cell proliferation in pancreatic cancer. Cancer Cell Int.

[41] McDonald PC, Chafe SC, Brown WS, Saberi S, Swayampakula M, Venkateswaran G (2019). Regulation of pH by carbonic anhydrase 9 mediates survival of pancreatic cancer cells with activated KRAS in response to hypoxia. Gastroenterology.

[42] Jones S, Zhang X, Parsons DW, Lin JC-H, Leary RJ, Angenendt P, et al. Core signaling pathways in human pancreatic cancers revealed by global genomic analyses. Science (New York, NY). 2008;321:1801–6. 10.1126/science.1164368 PMC2848990 18772397

[43] Xiang J, Hu Q, Qin Y, Ji S, Xu W, Liu W (2018). TCF7L2 positively regulates aerobic glycolysis via the EGLN2/HIF-1alpha axis and indicates prognosis in pancreatic cancer. Cell Death Dis.

[44] Zhang Q, Lou Y, Zhang J, Fu Q, Wei T, Sun X (2017). Hypoxia-inducible factor-2alpha promotes tumor progression and has crosstalk with Wnt/beta-catenin signaling in pancreatic cancer. Mol Cancer.

[45] Ren H, Zhao T, Sun J, Wang X, Liu J, Gao S (2013). The CX3CL1/CX3CR1 reprograms glucose metabolism through HIF-1 pathway in pancreatic adenocarcinoma. J Cell Biochem.

[46] Yang J, Wang F, Chen X, Qiu S, Cui L, Hu L (2019). beta-pentagalloyl-glucose sabotages pancreatic cancer cells and ameliorates cachexia in tumor-bearing mice. Am J Chin Med.

[47] Li X, Truty MA, Kang Ya, Chopin-Laly X, Zhang R, Roife D, et al. Extracellular lumican inhibits pancreatic cancer cell growth and is associated with prolonged survival after surgery. Clin Cancer Res Off J Am Assoc Cancer Res 2014;20:6529–6540. 10.1158/1078-0432.CCR-14-0970 PMC4268437 25336691

[48] Bronner C, Krifa M, Mousli M (2013). Increasing role of UHRF1 in the reading and inheritance of the epigenetic code as well as in tumorogenesis. Biochem Pharmacol.

[49] Hashimoto H, Horton JR, Zhang X, Cheng X (2009). UHRF1, a modular multi-domain protein, regulates replication-coupled crosstalk between DNA methylation and histone modifications. Epigenetics.

[50] Cui XG, Han ZT, He SH, Wu XD, Chen TR, Shao CH (2017). HIF1/2alpha mediates hypoxia-induced LDHA expression in human pancreatic cancer cells. Oncotarget.

[51] Cui J, Quan M, Jiang W, Hu H, Jiao F, Li N (2015). Suppressed expression of LDHB promotes pancreatic cancer progression via inducing glycolytic phenotype. Med Oncol (Northwood, London, England).

[52] Feil R, Fraga MF (2012). Epigenetics and the environment: emerging patterns and implications. Nat Rev Genet.

[53] Wu DH, Liang H, Lu SN, Wang H, Su ZL, Zhang L (2018). miR-124 suppresses pancreatic ductal adenocarcinoma growth by regulating monocarboxylate transporter 1-mediated cancer lactate metabolism. Cell Physiol Biochem.

[54] Regel I, Kong B, Raulefs S, Erkan M, Michalski CW, Hartel M (2012). Energy metabolism and proliferation in pancreatic carcinogenesis. Langenbecks Arch Surg.

[55] Halbrook CJ, Lyssiotis CA (2017). Employing metabolism to improve the diagnosis and treatment of pancreatic cancer. Cancer Cell.

[56] Cassago A, Ferreira AP, Ferreira IM, Fornezari C, Gomes ER, Greene KS (2012). Mitochondrial localization and structure-based phosphate activation mechanism of Glutaminase C with implications for cancer metabolism. Proc Natl Acad Sci U S A.

[57] Son J, Lyssiotis CA, Ying H, Wang X, Hua S, Ligorio M (2013). Glutamine supports pancreatic cancer growth through a KRAS-regulated metabolic pathway. Nature.

[58] Lyssiotis CA, Son J, Cantley LC, Kimmelman AC (2013). Pancreatic cancers rely on a novel glutamine metabolism pathway to maintain redox balance. Cell Cycle.

[59] Ma Y, Li Y, Ling S, Li X, Kong B, Hu M (2020). Loss of heterozygosity for Kras promotes REDD1-dependent, non-canonical glutamine metabolism in pancreatic ductal adenocarcinoma. Biochem Biophys Res Commun.

[60] Yoo HC, Park SJ, Nam M, Kang J, Kim K, Yeo JH (2020). A variant of SLC1A5 Is a mitochondrial glutamine transporter for metabolic reprogramming in cancer cells. Cell Metab.

[61] Knudsen ES, Balaji U, Freinkman E, McCue P, Witkiewicz AK (2016). Unique metabolic features of pancreatic cancer stroma: relevance to the tumor compartment, prognosis, and invasive potential. Oncotarget.

[62] Elgogary A, Xu Q, Poore B, Alt J, Zimmermann SC, Zhao L (2016). Combination therapy with BPTES nanoparticles and metformin targets the metabolic heterogeneity of pancreatic cancer. Proc Natl Acad Sci USA.

[63] Metallo CM, Gameiro PA, Bell EL, Mattaini KR, Yang J, Hiller K (2011). Reductive glutamine metabolism by IDH1 mediates lipogenesis under hypoxia. Nature.

[64] Anastasiou D, Cantley LC (2012). Breathless cancer cells get fat on glutamine. Cell Res.

[65] Sun RC, Denko NC (2014). Hypoxic regulation of glutamine metabolism through HIF1 and SIAH2 supports lipid synthesis that is necessary for tumor growth. Cell Metab.

[66] Schug ZT, Peck B, Jones DT, Zhang Q, Grosskurth S, Alam IS (2015). Acetyl-CoA synthetase 2 promotes acetate utilization and maintains cancer cell growth under metabolic stress. Cancer Cell.

[67] Bulusu V, Tumanov S, Michalopoulou E, van den Broek NJ, MacKay G, Nixon C (2017). Acetate recapturing by nuclear acetyl-CoA synthetase 2 prevents loss of histone acetylation during oxygen and serum limitation. Cell Rep.

[68] Huang, Li T, Li X, Zhang L, Sun L, He X, et al. HIF-1-mediated suppression of acyl-CoA dehydrogenases and fatty acid oxidation is critical for cancer progression. Cell Rep. 2014;8:1930–1942. 10.1016/j.celrep.2014.08.028 25242319

[69] Kamphorst JJ, Cross JR, Fan J, de Stanchina E, Mathew R, White EP (2013). Hypoxic and Ras-transformed cells support growth by scavenging unsaturated fatty acids from lysophospholipids. Proc Natl Acad Sci U S A.

[70] Cao J, Li J, Sun L, Qin T, Xiao Y, Chen K (2019). Hypoxia-driven paracrine osteopontin/integrin αvβ3 signaling promotes pancreatic cancer cell epithelial-mesenchymal transition and cancer stem cell-like properties by modulating forkhead box protein M1. Mol Oncol.

[71] Fruehauf JP, Meyskens FL (2007). Reactive oxygen species: a breath of life or death?. Clin Cancer Res.

[72] Nogueira V, Park Y, Chen CC, Xu PZ, Chen ML, Tonic I (2008). Akt determines replicative senescence and oxidative or oncogenic premature senescence and sensitizes cells to oxidative apoptosis. Cancer Cell.

[73] Zhang J, Yang J, Lin C, Liu W, Huo Y, Yang M (2020). Endoplasmic reticulum stress-dependent expression of ERO1L promotes aerobic glycolysis in pancreatic cancer. Theranostics.

[74] Sendoel A, Hengartner MO (2014). Apoptotic cell death under hypoxia. Physiology (Bethesda).

[75] Calvani M, Comito G, Giannoni E, Chiarugi P (2012). Time-dependent stabilization of hypoxia inducible factor-1alpha by different intracellular sources of reactive oxygen species. PLoS ONE.

[76] Jung SN, Yang WK, Kim J, Kim HS, Kim EJ, Yun H (2008). Reactive oxygen species stabilize hypoxia-inducible factor-1 alpha protein and stimulate transcriptional activity via AMP-activated protein kinase in DU145 human prostate cancer cells. Carcinogenesis.

[77] Klimova T, Chandel NS (2008). Mitochondrial complex III regulates hypoxic activation of HIF. Cell Death Differ.

[78] Lu H, Dalgard CL, Mohyeldin A, McFate T, Tait AS, Verma A (2005). Reversible inactivation of HIF-1 prolyl hydroxylases allows cell metabolism to control basal HIF-1. J Biol Chem.

[79] Zhao T, Gao S, Wang X, Liu J, Duan Y, Yuan Z (2012). Hypoxia-inducible factor-1alpha regulates chemotactic migration of pancreatic ductal adenocarcinoma cells through directly transactivating the CX3CR1 gene. PLoS ONE.

[80] Kwon SJ, Song JJ, Lee YJ (2005). Signal pathway of hypoxia-inducible factor-1alpha phosphorylation and its interaction with von Hippel-Lindau tumor suppressor protein during ischemia in MiaPaCa-2 pancreatic cancer cells. Clin Cancer Res.

[81] Wang M, Kirk JS, Venkataraman S, Domann FE, Zhang HJ, Schafer FQ (2005). Manganese superoxide dismutase suppresses hypoxic induction of hypoxia-inducible factor-1alpha and vascular endothelial growth factor. Oncogene.

[82] Wilkes JG, O'Leary BR, Du J, Klinger AR, Sibenaller ZA, Doskey CM (2018). Pharmacologic ascorbate (P-AscH(-)) suppresses hypoxia-inducible Factor-1α (HIF-1α) in pancreatic adenocarcinoma. Clin Exp Metastasis.

[83] Bienvenu P, Caron L, Gasparutto D, Kergonou JF (1992). Assessing and counteracting the prooxidant effects of anticancer drugs. EXS.

[84] Dorie MJ, Kallman RF (1986). Reoxygenation of the RIF-1 tumor after fractionated radiotherapy. Int J Radiat Oncol Biol Phys.

[85] Li C, Jackson RM (2002). Reactive species mechanisms of cellular hypoxia-reoxygenation injury. Am J Physiol Cell Physiol.

[86] Hirai F, Motoori S, Kakinuma S, Tomita K, Indo HP, Kato H (2004). Mitochondrial signal lacking manganese superoxide dismutase failed to prevent cell death by reoxygenation following hypoxia in a human pancreatic cancer cell line, KP4. Antioxid Redox Signal.

[87] Binker MG, Binker-Cosen AA, Richards D, Gaisano HY, de Cosen RH, Cosen-Binker LI (2010). Hypoxia-reoxygenation increase invasiveness of PANC-1 cells through Rac1/MMP-2. Biochem Biophys Res Commun.

[88] Lei J, Huo X, Duan W, Xu Q, Li R, Ma J (2014). α-Mangostin inhibits hypoxia-driven ROS-induced PSC activation and pancreatic cancer cell invasion. Cancer Lett.

[89] Topalovski M, Hagopian M, Wang M, Brekken RA (2016). Hypoxia and transforming growth factor β cooperate to induce Fibulin-5 expression in pancreatic cancer. J Biol Chem.

[90] Rhim AD, Oberstein PE, Thomas DH, Mirek ET, Palermo CF, Sastra SA (2014). Stromal elements act to restrain, rather than support, pancreatic ductal adenocarcinoma. Cancer Cell.

[91] Wang M, Topalovski M, Toombs JE, Wright CM, Moore ZR, Boothman DA (2015). Fibulin-5 Blocks Microenvironmental ROS in Pancreatic Cancer. Cancer Res.

[92] Estaras M, Martinez-Morcillo S, García A, Martinez R, Estevez M, Perez-Lopez M (2020). Pancreatic stellate cells exhibit adaptation to oxidative stress evoked by hypoxia. Biol Cell.

[93] Abdalla MY, Ahmad IM, Rachagani S, Banerjee K, Thompson CM, Maurer HC (2019). Enhancing responsiveness of pancreatic cancer cells to gemcitabine treatment under hypoxia by heme oxygenase-1 inhibition. Transl Res.

[94] Fukuda R, Zhang H, Kim JW, Shimoda L, Dang CV, Semenza GL (2007). HIF-1 regulates cytochrome oxidase subunits to optimize efficiency of respiration in hypoxic cells. Cell.

[95] Kim JW, Tchernyshyov I, Semenza GL, Dang CV (2006). HIF-1-mediated expression of pyruvate dehydrogenase kinase: a metabolic switch required for cellular adaptation to hypoxia. Cell Metab.

[96] Lang M, Wang X, Wang H, Dong J, Lan C, Hao J (2016). Arsenic trioxide plus PX-478 achieves effective treatment in pancreatic ductal adenocarcinoma. Cancer Lett.

[97] Ropolo A, Catrinacio C, Renna FJ, Boggio V, Orquera T, Gonzalez CD (2020). A Novel E2F1-EP300-VMP1 Pathway mediates gemcitabine-induced autophagy in pancreatic cancer cells carrying oncogenic KRAS. Front Endocrinol (Lausanne).

[98] Ozpolat B, Akar U, Mehta K, Lopez-Berestein G (2007). PKC delta and tissue transglutaminase are novel inhibitors of autophagy in pancreatic cancer cells. Autophagy.

[99] Ma J, Weng L, Jia Y, Liu B, Wu S, Xue L (2020). PTBP3 promotes malignancy and hypoxia-induced chemoresistance in pancreatic cancer cells by ATG12 up-regulation. J Cell Mol Med.

[100] Joshi S, Kumar S, Ponnusamy MP, Batra SK (2016). Hypoxia-induced oxidative stress promotes MUC4 degradation via autophagy to enhance pancreatic cancer cells survival. Oncogene.

[101] Li X, Lee Y, Kang Y, Dai B, Perez MR, Pratt M (2019). Hypoxia-induced autophagy of stellate cells inhibits expression and secretion of lumican into microenvironment of pancreatic ductal adenocarcinoma. Cell Death Differ.

[102] Nikitovic D, Papoutsidakis A, Karamanos NK, Tzanakakis GN (2014). Lumican affects tumor cell functions, tumor-ECM interactions, angiogenesis and inflammatory response. Matrix Biol.

[103] Sahni S, Gillson J, Park KC, Chiang S, Leck LYW, Jansson PJ (2020). NDRG1 suppresses basal and hypoxia-induced autophagy at both the initiation and degradation stages and sensitizes pancreatic cancer cells to lysosomal membrane permeabilization. Biochim Biophys Acta Gen Subj.

[104] Tian S, Guo X, Yu C, Sun C, Jiang J (2017). miR-138-5p suppresses autophagy in pancreatic cancer by targeting SIRT1. Oncotarget.

[105] Maertin S, Elperin JM, Lotshaw E, Sendler M, Speakman SD, Takakura K (2017). Roles of autophagy and metabolism in pancreatic cancer cell adaptation to environmental challenges. Am J Physiol Gastrointest Liver Physiol.

[106] Hamidi T, Cano CE, Grasso D, Garcia MN, Sandi MJ, Calvo EL (2012). Nupr1-aurora kinase A pathway provides protection against metabolic stress-mediated autophagic-associated cell death. Clin Cancer Res Off J Am Assoc Cancer Res.

[107] Owada S, Ito K, Endo H, Shida Y, Okada C, Nezu T (2017). An adaptation system to avoid apoptosis via autophagy under hypoxic conditions in pancreatic cancer cells. Anticancer Res.

[108] Frieboes HB, Huang JS, Yin WC, McNally LR (2014). Chloroquine-mediated cell death in metastatic pancreatic adenocarcinoma through inhibition of autophagy. JOP.

[109] Hermann PC, Huber SL, Herrler T, Aicher A, Ellwart JW, Guba M (2007). Distinct populations of cancer stem cells determine tumor growth and metastatic activity in human pancreatic cancer. Cell Stem Cell.

[110] Hashimoto O, Shimizu K, Semba S, Chiba S, Ku Y, Yokozaki H (2011). Hypoxia induces tumor aggressiveness and the expansion of CD133-positive cells in a hypoxia-inducible factor-1α-dependent manner in pancreatic cancer cells. Pathobiology.

[111] Jaiswal KR, Xin HW, Anderson A, Wiegand G, Kim B, Miller T (2012). Comparative testing of various pancreatic cancer stem cells results in a novel class of pancreatic-cancer-initiating cells. Stem Cell Res.

[112] Li C, Heidt DG, Dalerba P, Burant CF, Zhang L, Adsay V (2007). Identification of pancreatic cancer stem cells. Cancer Res.

[113] Sergeant G, Vankelecom H, Gremeaux L, Topal B (2009). Role of cancer stem cells in pancreatic ductal adenocarcinoma. Nat Rev Clin Oncol.

[114] Kawamoto M, Ishiwata T, Cho K, Uchida E, Korc M, Naito Z (2009). Nestin expression correlates with nerve and retroperitoneal tissue invasion in pancreatic cancer. Hum Pathol.

[115] Li C, Wu JJ, Hynes M, Dosch J, Sarkar B, Welling TH (2011). c-Met is a marker of pancreatic cancer stem cells and therapeutic target. Gastroenterology.

[116] Maeda K, Ding Q, Yoshimitsu M, Kuwahata T, Miyazaki Y, Tsukasa K (2016). CD133 modulate HIF-1α expression under hypoxia in EMT Phenotype pancreatic cancer stem-like cells. Int J Mol Sci..

[117] Su HT, Weng CC, Hsiao PJ, Chen LH, Kuo TL, Chen YW (2013). Stem cell marker nestin is critical for TGF-β1-mediated tumor progression in pancreatic cancer. Mol Cancer Res.

[118] Mohyeldin A, Garzon-Muvdi T, Quinones-Hinojosa A (2010). Oxygen in stem cell biology: a critical component of the stem cell niche. Cell Stem Cell.

[119] Heddleston JM, Li Z, Lathia JD, Bao S, Hjelmeland AB, Rich JN (2010). Hypoxia inducible factors in cancer stem cells. Br J Cancer.

[120] Nomura A, Dauer P, Gupta V, McGinn O, Arora N, Majumdar K (2016). Microenvironment mediated alterations to metabolic pathways confer increased chemo-resistance in CD133+ tumor initiating cells. Oncotarget.

[121] Chen S, Zhang J, Chen J, Wang Y, Zhou S, Huang L (2019). RER1 enhances carcinogenesis and stemness of pancreatic cancer under hypoxic environment. J Exp Clin Cancer Res.

[122] McGinn O, Gupta VK, Dauer P, Arora N, Sharma N, Nomura A (2017). Inhibition of hypoxic response decreases stemness and reduces tumorigenic signaling due to impaired assembly of HIF1 transcription complex in pancreatic cancer. Sci Rep.

[123] Zhu H, Wang D, Zhang L, Xie X, Wu Y, Liu Y (2014). Upregulation of autophagy by hypoxia-inducible factor-1α promotes EMT and metastatic ability of CD133+ pancreatic cancer stem-like cells during intermittent hypoxia. Oncol Rep.

[124] Zhu H, Wang D, Liu Y, Su Z, Zhang L, Chen F (2013). Role of the Hypoxia-inducible factor-1 alpha induced autophagy in the conversion of non-stem pancreatic cancer cells into CD133+ pancreatic cancer stem-like cells. Cancer Cell Int.

[125] Rausch V, Liu L, Apel A, Rettig T, Gladkich J, Labsch S (2012). Autophagy mediates survival of pancreatic tumour-initiating cells in a hypoxic microenvironment. J Pathol.

[126] Peng G, Tang Z, Xiang Y, Chen W (2019). Glutathione peroxidase 4 maintains a stemness phenotype, oxidative homeostasis and regulates biological processes in Panc-1 cancer stem-like cells. Oncol Rep.

[127] De Bock K, Mazzone M, Carmeliet P (2011). Antiangiogenic therapy, hypoxia, and metastasis: risky liaisons, or not?. Nat Rev Clin Oncol.

[128] Chen S, Chen X, Li W, Shan T, Lin WR, Ma J (2018). Conversion of epithelial-to-mesenchymal transition to mesenchymal-to-epithelial transition is mediated by oxygen concentration in pancreatic cancer cells. Oncol Lett.

[129] Yang MH, Wu MZ, Chiou SH, Chen PM, Chang SY, Liu CJ (2008). Direct regulation of TWIST by HIF-1alpha promotes metastasis. Nat Cell Biol.

[130] Julien S, Puig I, Caretti E, Bonaventure J, Nelles L, van Roy F (2007). Activation of NF-kappaB by Akt upregulates Snail expression and induces epithelium mesenchyme transition. Oncogene.

[131] Chua HL, Bhat-Nakshatri P, Clare SE, Morimiya A, Badve S, Nakshatri H (2007). NF-kappaB represses E-cadherin expression and enhances epithelial to mesenchymal transition of mammary epithelial cells: potential involvement of ZEB-1 and ZEB-2. Oncogene.

[132] Ni J, Zhou S, Yuan W, Cen F, Yan Q (2019). Mechanism of miR-210 involved in epithelial-mesenchymal transition of pancreatic cancer cells under hypoxia. J Recept Signal Transduct Res.

[133] Cheng ZX, Wang DW, Liu T, Liu WX, Xia WB, Xu J (2014). Effects of the HIF-1α and NF-κB loop on epithelial-mesenchymal transition and chemoresistance induced by hypoxia in pancreatic cancer cells. Oncol Rep.

[134] Shimojo Y, Akimoto M, Hisanaga T, Tanaka T, Tajima Y, Honma Y (2013). Attenuation of reactive oxygen species by antioxidants suppresses hypoxia-induced epithelial-mesenchymal transition and metastasis of pancreatic cancer cells. Clin Exp Metastasis.

[135] Liu L, Salnikov AV, Bauer N, Aleksandrowicz E, Labsch S, Nwaeburu C (2014). Triptolide reverses hypoxia-induced epithelial-mesenchymal transition and stem-like features in pancreatic cancer by NF-κB downregulation. Int J Cancer.

[136] Hotz B, Arndt M, Dullat S, Bhargava S, Buhr HJ, Hotz HG (2007). Epithelial to mesenchymal transition: expression of the regulators snail, slug, and twist in pancreatic cancer. Clin Cancer Res.

[137] Chen S, Chen JZ, Zhang JQ, Chen HX, Yan ML, Huang L (2016). Hypoxia induces TWIST-activated epithelial-mesenchymal transition and proliferation of pancreatic cancer cells in vitro and in nude mice. Cancer Lett.

[138] Yang J, Zhang X, Zhang Y, Zhu D, Zhang L, Li Y (2016). HIF-2α promotes epithelial-mesenchymal transition through regulating Twist2 binding to the promoter of E-cadherin in pancreatic cancer. J Exp Clin Cancer Res.

[139] Ikenouchi J, Matsuda M, Furuse M, Tsukita S (2003). Regulation of tight junctions during the epithelium-mesenchyme transition: direct repression of the gene expression of claudins/occludin by Snail. J Cell Sci.

[140] Kyuno D, Kojima T, Yamaguchi H, Ito T, Kimura Y, Imamura M (2013). Protein kinase Cα inhibitor protects against downregulation of claudin-1 during epithelial-mesenchymal transition of pancreatic cancer. Carcinogenesis.

[141] Kojima T, Takasawa A, Kyuno D, Ito T, Yamaguchi H, Hirata K (2011). Downregulation of tight junction-associated MARVEL protein marvelD3 during epithelial-mesenchymal transition in human pancreatic cancer cells. Exp Cell Res.

[142] Kizaka-Kondoh S, Itasaka S, Zeng L, Tanaka S, Zhao T, Takahashi Y (2009). Selective killing of hypoxia-inducible factor-1-active cells improves survival in a mouse model of invasive and metastatic pancreatic cancer. Clin Cancer Res.

[143] Chiou SH, Risca VI, Wang GX, Yang D, Grüner BM, Kathiria AS (2017). BLIMP1 Induces Transient Metastatic Heterogeneity in Pancreatic Cancer. Cancer Discov.

[144] Wei H, Li F, Fu P, Liu X (2013). Effects of the silencing of hypoxia-inducible Factor-1 alpha on metastasis of pancreatic cancer. Eur Rev Med Pharmacol Sci.

[145] Zeng Z, Lei S, He Z, Chen T, Jiang J (2020). YEATS2 is a target of HIF1α and promotes pancreatic cancer cell proliferation and migration. J Cell Physiol.

[146] Büchler P, Reber HA, Tomlinson JS, Hankinson O, Kallifatidis G, Friess H (2009). Transcriptional regulation of urokinase-type plasminogen activator receptor by hypoxia-inducible factor 1 is crucial for invasion of pancreatic and liver cancer. Neoplasia.

[147] Zhao T, Ren H, Li J, Chen J, Zhang H, Xin W (2015). LASP1 is a HIF1α target gene critical for metastasis of pancreatic cancer. Cancer Res.

[148] Shi CY, Fan Y, Liu B, Lou WH (2013). HIF1 contributes to hypoxia-induced pancreatic cancer cells invasion via promoting QSOX1 expression. Cell Physiol Biochem.

[149] Zhou X, Guo X, Chen M, Xie C, Jiang J (2018). HIF-3α Promotes metastatic phenotypes in pancreatic cancer by transcriptional regulation of the RhoC-ROCK1 signaling pathway. Mol Cancer Res.

[150] Miller BW, Morton JP, Pinese M, Saturno G, Jamieson NB, McGhee E (2015). Targeting the LOX/hypoxia axis reverses many of the features that make pancreatic cancer deadly: inhibition of LOX abrogates metastasis and enhances drug efficacy. EMBO Mol Med.

[151] Manoli S, Coppola S, Duranti C, Lulli M, Magni L, Kuppalu N (2019). The activity of Kv 11.1 potassium channel modulates F-actin organization during cell migration of pancreatic ductal adenocarcinoma cells. Cancers (Basel).

[152] Tiwari A, Tashiro K, Dixit A, Soni A, Vogel K, Hall B (2020). Loss of HIF1A from pancreatic cancer cells increases expression of ppp1r1b and degradation of p53 to promote invasion and metastasis. Gastroenterology.

[153] Lee KE, Spata M, Bayne LJ, Buza EL, Durham AC, Allman D (2016). Hif1a deletion reveals pro-neoplastic function of B cells in pancreatic neoplasia. Cancer Discov.

[154] Leppanen J, Helminen O, Huhta H, Kauppila JH, Isohookana J, Haapasaari KM (2018). Weak HIF-1alpha expression indicates poor prognosis in resectable pancreatic ductal adenocarcinoma. World J Surg Oncol.

[155] Inaguma S, Kasai K, Ikeda H (2011). GLI1 facilitates the migration and invasion of pancreatic cancer cells through MUC5AC-mediated attenuation of E-cadherin. Oncogene.

[156] Bailey JM, Mohr AM, Hollingsworth MA (2009). Sonic hedgehog paracrine signaling regulates metastasis and lymphangiogenesis in pancreatic cancer. Oncogene.

[157] Lei J, Ma J, Ma Q, Li X, Liu H, Xu Q (2013). Hedgehog signaling regulates hypoxia induced epithelial to mesenchymal transition and invasion in pancreatic cancer cells via a ligand-independent manner. Mol Cancer.

[158] Cao L, Xiao X, Lei J, Duan W, Ma Q, Li W (2016). Curcumin inhibits hypoxia-induced epithelial-mesenchymal transition in pancreatic cancer cells via suppression of the hedgehog signaling pathway. Oncol Rep.

[159] Li W, Cao L, Chen X, Lei J, Ma Q (2016). Resveratrol inhibits hypoxia-driven ROS-induced invasive and migratory ability of pancreatic cancer cells via suppression of the Hedgehog signaling pathway. Oncol Rep.

[160] Onishi H, Morisaki T, Nakao F, Odate S, Morisaki T, Katano M (2013). Protein-bound polysaccharide decreases invasiveness and proliferation in pancreatic cancer by inhibition of hedgehog signaling and HIF-1α pathways under hypoxia. Cancer Lett.

[161] Morifuji Y, Onishi H, Iwasaki H, Imaizumi A, Nakano K, Tanaka M (2014). Reoxygenation from chronic hypoxia promotes metastatic processes in pancreatic cancer through the Hedgehog signaling. Cancer Sci.

[162] Sanchez-Tillo E, de Barrios O, Siles L, Cuatrecasas M, Castells A, Postigo A (2011). beta-catenin/TCF4 complex induces the epithelial-to-mesenchymal transition (EMT)-activator ZEB1 to regulate tumor invasiveness. Proc Natl Acad Sci U S A.

[163] Guo Q, Qin W (2015). DKK3 blocked translocation of β-catenin/EMT induced by hypoxia and improved gemcitabine therapeutic effect in pancreatic cancer Bxpc-3 cell. J Cell Mol Med.

[164] Wu DJ, Jiang YS, He RZ, Tao LY, Yang MW, Fu XL (2018). High expression of WNT7A predicts poor prognosis and promote tumor metastasis in pancreatic ductal adenocarcinoma. Sci Rep.

[165] Li H, Wang X, Wen C, Huo Z, Wang W, Zhan Q (2017). Long noncoding RNA NORAD, a novel competing endogenous RNA, enhances the hypoxia-induced epithelial-mesenchymal transition to promote metastasis in pancreatic cancer. Mol Cancer.

[166] Zhang KD, Hu B, Cen G, Yang YH, Chen WW, Guo ZY (2020). MiR-301a transcriptionally activated by HIF-2α promotes hypoxia-induced epithelial-mesenchymal transition by targeting TP63 in pancreatic cancer. World J Gastroenterol.

[167] Ou ZL, Zhang M, Ji LD, Luo Z, Han T, Lu YB (2019). Long noncoding RNA FEZF1-AS1 predicts poor prognosis and modulates pancreatic cancer cell proliferation and invasion through miR-142/HIF-1α and miR-133a/EGFR upon hypoxia/normoxia. J Cell Physiol.

[168] Zhu G, Zhou L, Liu H, Shan Y, Zhang X (2018). MicroRNA-224 promotes pancreatic cancer cell proliferation and migration by targeting the TXNIP-mediated HIF1α pathway. Cell Physiol Biochem.

[169] Wang X, Luo G, Zhang K, Cao J, Huang C, Jiang T (2018). Hypoxic tumor-derived exosomal miR-301a mediates M2 macrophage polarization via PTEN/PI3Kγ to promote pancreatic cancer metastasis. Cancer Res.

[170] Pan L, Zhou L, Yin W, Bai J, Liu R (2018). miR-125a induces apoptosis, metabolism disorder and migrationimpairment in pancreatic cancer cells by targeting Mfn2-related mitochondrial fission. Int J Oncol.

[171] Schmitt AM, Chang HY (2016). Long noncoding RNAs in cancer pathways. Cancer Cell.

[172] Chen SY, Teng SC, Cheng TH, Wu KJ (2016). miR-1236 regulates hypoxia-induced epithelial-mesenchymal transition and cell migration/invasion through repressing SENP1 and HDAC3. Cancer Lett.

[173] Luo G, Xia X, Wang X, Zhang K, Cao J, Jiang T (2018). miR-301a plays a pivotal role in hypoxia-induced gemcitabine resistance in pancreatic cancer. Exp Cell Res.

[174] Zeng Z, Xu FY, Zheng H, Cheng P, Chen QY, Ye Z (2019). LncRNA-MTA2TR functions as a promoter in pancreatic cancer via driving deacetylation-dependent accumulation of HIF-1α. Theranostics.

[175] Deng SJ, Chen HY, Ye Z, Deng SC, Zhu S, Zeng Z (2018). Hypoxia-induced LncRNA-BX111 promotes metastasis and progression of pancreatic cancer through regulating ZEB1 transcription. Oncogene.

[176] Li X, Deng SJ, Zhu S, Jin Y, Cui SP, Chen JY (2016). Hypoxia-induced lncRNA-NUTF2P3-001 contributes to tumorigenesis of pancreatic cancer by derepressing the miR-3923/KRAS pathway. Oncotarget.

[177] Liu M, Zhong J, Zeng Z, Huang K, Ye Z, Deng S (2019). Hypoxia-induced feedback of HIF-1α and lncRNA-CF129 contributes to pancreatic cancer progression through stabilization of p53 protein. Theranostics.

[178] Ou ZL, Luo Z, Wei W, Liang S, Gao TL, Lu YB (2019). Hypoxia-induced shedding of MICA and HIF1A-mediated immune escape of pancreatic cancer cells from NK cells: role of circ_0000977/miR-153 axis. RNA Biol.

[179] Song Z, Ren H, Gao S, Zhao X, Zhang H, Hao J (2014). The clinical significance and regulation mechanism of hypoxia-inducible factor-1 and miR-191 expression in pancreatic cancer. Tumour Biol.

[180] Sun J, Jiang Z, Li Y, Wang K, Chen X, Liu G (2019). Downregulation of miR-21 inhibits the malignant phenotype of pancreatic cancer cells by targeting VHL. Onco Targets Ther.

[181] Mace TA, Collins AL, Wojcik SE, Croce CM, Lesinski GB, Bloomston M (2013). Hypoxia induces the overexpression of microRNA-21 in pancreatic cancer cells. J Surg Res.

[182] Yue H, Liu L, Song Z (2019). miR-212 regulated by HIF-1α promotes the progression of pancreatic cancer. Exp Ther Med.

[183] Niu Y, Jin Y, Deng SC, Deng SJ, Zhu S, Liu Y (2018). MiRNA-646-mediated reciprocal repression between HIF-1α and MIIP contributes to tumorigenesis of pancreatic cancer. Oncogene.

[184] Lu Y, Ji N, Wei W, Sun W, Gong X, Wang X (2017). MiR-142 modulates human pancreatic cancer proliferation and invasion by targeting hypoxia-inducible factor 1 (HIF-1α) in the tumor microenvironments. Biol Open.

[185] Sun JS, Zhang XL, Yang YJ, Nie ZG, Zhang Y (2015). Hypoxia promotes C-X-C chemokine receptor type 4 expression through microRNA-150 in pancreatic cancer cells. Oncol Lett.

[186] Nong K, Zhang D, Chen C, Yang Y, Yang Y, Liu S (2020). MicroRNA-519 inhibits hypoxia-induced tumorigenesis of pancreatic cancer by regulating immune checkpoint PD-L1. Oncol Lett.

[187] Zhu S, He C, Deng S, Li X, Cui S, Zeng Z (2016). MiR-548an, transcriptionally downregulated by HIF1α/HDAC1, suppresses tumorigenesis of pancreatic cancer by targeting vimentin expression. Mol Cancer Ther.

[188] Salnikov AV, Liu L, Platen M, Gladkich J, Salnikova O, Ryschich E (2012). Hypoxia induces EMT in low and highly aggressive pancreatic tumor cells but only cells with cancer stem cell characteristics acquire pronounced migratory potential. PLoS ONE.

[189] Ding Q, Miyazaki Y, Tsukasa K, Matsubara S, Yoshimitsu M, Takao S (2014). CD133 facilitates epithelial-mesenchymal transition through interaction with the ERK pathway in pancreatic cancer metastasis. Mol Cancer.

[190] Ide T, Kitajima Y, Miyoshi A, Ohtsuka T, Mitsuno M, Ohtaka K (2006). Tumor-stromal cell interaction under hypoxia increases the invasiveness of pancreatic cancer cells through the hepatocyte growth factor/c-Met pathway. Int J Cancer.

[191] Sada M, Ohuchida K, Horioka K, Okumura T, Moriyama T, Miyasaka Y (2016). Hypoxic stellate cells of pancreatic cancer stroma regulate extracellular matrix fiber organization and cancer cell motility. Cancer Lett.

[192] Li W, Sun L, Lei J, Wu Z, Ma Q, Wang Z (2020). Curcumin inhibits pancreatic cancer cell invasion and EMT by interfering with tumor-stromal crosstalk under hypoxic conditions via the IL-6/ERK/NF-κB axis. Oncol Rep.

[193] Özdemir BC, Pentcheva-Hoang T, Carstens JL, Zheng X, Wu CC, Simpson TR (2014). Depletion of carcinoma-associated fibroblasts and fibrosis induces immunosuppression and accelerates pancreas cancer with reduced survival. Cancer Cell.

[194] Ino Y, Yamazaki-Itoh R, Oguro S, Shimada K, Kosuge T, Zavada J (2013). Arginase II expressed in cancer-associated fibroblasts indicates tissue hypoxia and predicts poor outcome in patients with pancreatic cancer. PLoS ONE.

[195] Tung KH, Lin CW, Kuo CC, Li LT, Kuo YH, Lin CW (2013). CHC promotes tumor growth and angiogenesis through regulation of HIF-1α and VEGF signaling. Cancer Lett.

[196] Su Y, Loos M, Giese N, Metzen E, Büchler MW, Friess H (2012). Prolyl hydroxylase-2 (PHD2) exerts tumor-suppressive activity in pancreatic cancer. Cancer.

[197] Su Y, Loos M, Giese N, Hines OJ, Diebold I, Görlach A (2010). PHD3 regulates differentiation, tumour growth and angiogenesis in pancreatic cancer. Br J Cancer.

[198] Shi Q, Le X, Abbruzzese JL, Wang B, Mujaida N, Matsushima K (1999). Cooperation between transcription factor AP-1 and NF-kappaB in the induction of interleukin-8 in human pancreatic adenocarcinoma cells by hypoxia. J Interferon Cytokine Res.

[199] Azoitei N, Becher A, Steinestel K, Rouhi A, Diepold K, Genze F (2016). PKM2 promotes tumor angiogenesis by regulating HIF-1α through NF-κB activation. Molecular cancer.

[200] Nagaraju GP, Zhu S, Ko JE, Ashritha N, Kandimalla R, Snyder JP (2015). Antiangiogenic effects of a novel synthetic curcumin analogue in pancreatic cancer. Cancer Lett.

[201] Wen Z, Huang C, Xu Y, Xiao Y, Tang L, Dai J (2016). α-Solanine inhibits vascular endothelial growth factor expression by down-regulating the ERK1/2-HIF-1α and STAT3 signaling pathways. Eur J Pharmacol.

[202] Niu F, Li Y, Lai FF, Ni L, Ji M, Jin J (2015). LB-1 Exerts Antitumor Activity in Pancreatic Cancer by Inhibiting HIF-1α and Stat3 Signaling. J Cell Physiol.

[203] Zou G-M, Karikari C, Kabe Y, Handa H, Anders RA, Maitra A (2009). The Ape-1/Ref-1 redox antagonist E3330 inhibits the growth of tumor endothelium and endothelial progenitor cells: therapeutic implications in tumor angiogenesis. J Cell Physiol.

[204] Sahraei M, Roy LD, Curry JM, Teresa TL, Nath S, Besmer D (2012). MUC1 regulates PDGFA expression during pancreatic cancer progression. Oncogene.

[205] Kitamoto S, Yokoyama S, Higashi M, Yamada N, Takao S, Yonezawa S (2013). MUC1 enhances hypoxia-driven angiogenesis through the regulation of multiple proangiogenic factors. Oncogene.

[206] Maruggi M, Layng FI, Lemos R, Garcia G, James BP, Sevilla M (2019). Absence of HIF1A Leads to Glycogen Accumulation and an Inflammatory Response That Enables Pancreatic Tumor Growth. Cancer Res.

[207] Chang LH, Pan SL, Lai CY, Tsai AC, Teng CM (2013). Activated PAR-2 regulates pancreatic cancer progression through ILK/HIF-α-induced TGF-α expression and MEK/VEGF-A-mediated angiogenesis. Am J Pathol.

[208] Wang Z, Li Y, Ahmad A, Banerjee S, Azmi AS, Kong D (2011). Pancreatic cancer: understanding and overcoming chemoresistance. Nat Rev Gastroenterol Hepatol.

[209] Wang Y, Kuramitsu Y, Tokuda K, Baron B, Kitagawa T, Akada J (2014). Gemcitabine induces poly (ADP-ribose) polymerase-1 (PARP-1) degradation through autophagy in pancreatic cancer. PLoS ONE.

[210] Altan B, Kaira K, Watanabe A, Kubo N, Bao P, Dolgormaa G (2018). Relationship between LAT1 expression and resistance to chemotherapy in pancreatic ductal adenocarcinoma. Cancer Chemother Pharmacol.

[211] Blanco FF, Jimbo M, Wulfkuhle J, Gallagher I, Deng J, Enyenihi L (2016). The mRNA-binding protein HuR promotes hypoxia-induced chemoresistance through posttranscriptional regulation of the proto-oncogene PIM1 in pancreatic cancer cells. Oncogene.

[212] Yokoi K, Fidler IJ (2004). Hypoxia increases resistance of human pancreatic cancer cells to apoptosis induced by gemcitabine. Clin Cancer Res.

[213] Singh NS, Bernier M, Wainer IW (2016). Selective GPR55 antagonism reduces chemoresistance in cancer cells. Pharmacol Res.

[214] He X, Wang J, Wei W, Shi M, Xin B, Zhang T (2016). Hypoxia regulates ABCG2 activity through the activivation of ERK1/2/HIF-1α and contributes to chemoresistance in pancreatic cancer cells. Cancer Biol Ther.

[215] Kasuya K, Tsuchida A, Nagakawa Y, Suzuki M, Abe Y, Itoi T (2011). Hypoxia-inducible factor-1α expression and gemcitabine chemotherapy for pancreatic cancer. Oncol Rep.

[216] Zhang X, Kumstel S, Jiang K, Meng S, Gong P, Vollmar B (2019). LW6 enhances chemosensitivity to gemcitabine and inhibits autophagic flux in pancreatic cancer. J Adv Res.

[217] Zhao T, Jin F, Xiao D, Wang H, Huang C, Wang X (2020). IL-37/ STAT3/ HIF-1α negative feedback signaling drives gemcitabine resistance in pancreatic cancer. Theranostics.

[218] Nagaraju GP, Zakka KM, Landry JC, Shaib WL, Lesinski GB, El-Rayes BF (2019). Inhibition of HSP90 overcomes resistance to chemotherapy and radiotherapy in pancreatic cancer. Int J Cancer.

[219] Tang LR, Wu JX, Cai SL, Huang YX, Zhang XQ, Fu WK (2018). Prolyl hydroxylase domain 3 influences the radiotherapy efficacy of pancreatic cancer cells by targeting hypoxia-inducible factor-1α. Onco Targets Ther.

[220] Arora S, Bhardwaj A, Singh S, Srivastava SK, McClellan S, Nirodi CS (2013). An undesired effect of chemotherapy: gemcitabine promotes pancreatic cancer cell invasiveness through reactive oxygen species-dependent, nuclear factor κB- and hypoxia-inducible factor 1α-mediated up-regulation of CXCR4. J Biol Chem.

[221] Qanungo S, Uys JD, Manevich Y, Distler AM, Shaner B, Hill EG (2014). N-acetyl-L-cysteine sensitizes pancreatic cancers to gemcitabine by targeting the NFκB pathway. Biomed Pharmacother.

[222] Wang L, Bi R, Yin H, Liu H, Li L (2019). ENO1 silencing impaires hypoxia-induced gemcitabine chemoresistance associated with redox modulation in pancreatic cancer cells. Am J Transl Res.

[223] Onishi H, Morifuji Y, Kai M, Suyama K, Iwasaki H, Katano M (2012). Hedgehog inhibitor decreases chemosensitivity to 5-fluorouracil and gemcitabine under hypoxic conditions in pancreatic cancer. Cancer Sci.

[224] Jain RK (2014). Antiangiogenesis strategies revisited: from starving tumors to alleviating hypoxia. Cancer Cell.

[225] Gioelli N, Maione F, Camillo C, Ghitti M, Valdembri D, Morello N (2018). A rationally designed NRP1-independent superagonist SEMA3A mutant is an effective anticancer agent. Sci Transl Med..

[226] Gilles ME, Maione F, Cossutta M, Carpentier G, Caruana L, Di Maria S (2016). Nucleolin targeting impairs the progression of pancreatic cancer and promotes the normalization of tumor vasculature. Cancer Res.

[227] Raykov Z, Grekova SP, Bour G, Lehn JM, Giese NA, Nicolau C (2014). Myo-inositol trispyrophosphate-mediated hypoxia reversion controls pancreatic cancer in rodents and enhances gemcitabine efficacy. Int J Cancer.

[228] Sasajima J, Mizukami Y, Sugiyama Y, Nakamura K, Kawamoto T, Koizumi K (2010). Transplanting normal vascular proangiogenic cells to tumor-bearing mice triggers vascular remodeling and reduces hypoxia in tumors. Cancer Res.

[229] Bai X, Zhi X, Zhang Q, Liang F, Chen W, Liang C (2014). Inhibition of protein phosphatase 2A sensitizes pancreatic cancer to chemotherapy by increasing drug perfusion via HIF-1α-VEGF mediated angiogenesis. Cancer Lett.

[230] Provenzano PP, Cuevas C, Chang AE, Goel VK, Von Hoff DD, Hingorani SR (2012). Enzymatic targeting of the stroma ablates physical barriers to treatment of pancreatic ductal adenocarcinoma. Cancer Cell.

[231] Li X, Shepard HM, Cowell JA, Zhao C, Osgood RJ, Rosengren S (2018). Parallel accumulation of tumor hyaluronan, collagen, and other drivers of tumor progression. Clin Cancer Res.

[232] Chauhan VP, Martin JD, Liu H, Lacorre DA, Jain SR, Kozin SV (2013). Angiotensin inhibition enhances drug delivery and potentiates chemotherapy by decompressing tumour blood vessels. Nat Commun.

[233] Cham KK, Baker JH, Takhar KS, Flexman JA, Wong MQ, Owen DA (2010). Metronomic gemcitabine suppresses tumour growth, improves perfusion, and reduces hypoxia in human pancreatic ductal adenocarcinoma. Br J Cancer.

[234] Yapp DT, Wong MQ, Kyle AH, Valdez SM, Tso J, Yung A (2016). The differential effects of metronomic gemcitabine and antiangiogenic treatment in patient-derived xenografts of pancreatic cancer: treatment effects on metabolism, vascular function, cell proliferation, and tumor growth. Angiogenesis.

[235] Zhang K, Fang Y, He Y, Yin H, Guan X, Pu Y (2019). Extravascular gelation shrinkage-derived internal stress enables tumor starvation therapy with suppressed metastasis and recurrence. Nat Commun.

[236] Kutluk Cenik B, Ostapoff KT, Gerber DE, Brekken RA (2013). BIBF 1120 (nintedanib), a triple angiokinase inhibitor, induces hypoxia but not EMT and blocks progression of preclinical models of lung and pancreatic cancer. Mol Cancer Ther.

[237] Ostapoff KT, Awasthi N, Cenik BK, Hinz S, Dredge K, Schwarz RE (2013). PG545, an angiogenesis and heparanase inhibitor, reduces primary tumor growth and metastasis in experimental pancreatic cancer. Mol Cancer Ther.

[238] Zhang Y, Kirane A, Huang H, Sorrelle NB, Burrows FJ, Dellinger MT (2019). Cyclooxygenase-2 Inhibition Potentiates the Efficacy of Vascular Endothelial Growth Factor Blockade and Promotes an Immune Stimulatory Microenvironment in Preclinical Models of Pancreatic Cancer. Mol Cancer Res.

[239] Gao Y, Yu X, Zhang F, Dai J (2019). Propofol inhibits pancreatic cancer progress under hypoxia via ADAM8. J Hepatobiliary Pancreat Sci.

[240] Miyake K, Shimada M, Nishioka M, Sugimoto K, Batmunkh E, Uto Y (2008). The novel hypoxic cell radiosensitizer, TX-1877 has antitumor activity through suppression of angiogenesis and inhibits liver metastasis on xenograft model of pancreatic cancer. Cancer Lett.

[241] Shin SW, Jung W, Choi C, Kim SY, Son A, Kim H (2018). Fucoidan-Manganese Dioxide Nanoparticles Potentiate Radiation Therapy by Co-Targeting Tumor Hypoxia and Angiogenesis. Mar Drugs..

[242] Hanahan D, Weinberg RA (2011). Hallmarks of cancer: the next generation. Cell.

[243] Lalani AS, Alters SE, Wong A, Albertella MR, Cleland JL, Henner WD (2007). Selective tumor targeting by the hypoxia-activated prodrug AQ4N blocks tumor growth and metastasis in preclinical models of pancreatic cancer. Clin Cancer Res.

[244] Lohse I, Rasowski J, Cao P, Pintilie M, Do T, Tsao MS (2016). Targeting hypoxic microenvironment of pancreatic xenografts with the hypoxia-activated prodrug TH-302. Oncotarget.

[245] Bailey KM, Cornnell HH, Ibrahim-Hashim A, Wojtkowiak JW, Hart CP, Zhang X (2014). Evaluation of the "steal" phenomenon on the efficacy of hypoxia activated prodrug TH-302 in pancreatic cancer. PLoS ONE.

[246] Wojtkowiak JW, Cornnell HC, Matsumoto S, Saito K, Takakusagi Y, Dutta P (2015). Pyruvate sensitizes pancreatic tumors to hypoxia-activated prodrug TH-302. Cancer & metabolism.

[247] Kulkarni P, Haldar MK, Katti P, Dawes C, You S, Choi Y (2016). Hypoxia Responsive, Tumor Penetrating Lipid Nanoparticles for Delivery of Chemotherapeutics to Pancreatic Cancer Cell Spheroids. Bioconjug Chem.

[248] Miyake K, Nishioka M, Imura S, Batmunkh E, Uto Y, Nagasawa H (2012). The novel hypoxic cytotoxin, TX-2098 has antitumor effect in pancreatic cancer; possible mechanism through inhibiting VEGF and hypoxia inducible factor-1alpha targeted gene expression. Exp Cell Res.

[249] Xu XL, Yang YR, Mo XF, Wei JL, Zhang XJ, You QD (2017). Design, synthesis, and evaluation of benzofuran derivatives as novel anti-pancreatic carcinoma agents via interfering the hypoxia environment by targeting HIF-1α pathway. Eur J Med Chem.

[250] Li M, Xie H, Liu Y, Xia C, Cun X, Long Y (2019). Knockdown of hypoxia-inducible factor-1 alpha by tumor targeted delivery of CRISPR/Cas9 system suppressed the metastasis of pancreatic cancer. J Control Release Off J Control Release Soc.

[251] Zhao X, Li F, Li Y, Wang H, Ren H, Chen J (2015). Co-delivery of HIF1α siRNA and gemcitabine via biocompatible lipid-polymer hybrid nanoparticles for effective treatment of pancreatic cancer. Biomaterials.

[252] Zhao T, Ren H, Jia L, Chen J, Xin W, Yan F (2015). Inhibition of HIF-1α by PX-478 enhances the anti-tumor effect of gemcitabine by inducing immunogenic cell death in pancreatic ductal adenocarcinoma. Oncotarget.

[253] Schwartz DL, Bankson JA, Lemos R, Lai SY, Thittai AK, He Y (2010). Radiosensitization and stromal imaging response correlates for the HIF-1 inhibitor PX-478 given with or without chemotherapy in pancreatic cancer. Mol Cancer Ther.

[254] Le A, Cooper CR, Gouw AM, Dinavahi R, Maitra A, Deck LM (2010). Inhibition of lactate dehydrogenase A induces oxidative stress and inhibits tumor progression. Proc Natl Acad Sci USA.

[255] Maftouh M, Avan A, Sciarrillo R, Granchi C, Leon LG, Rani R (2014). Synergistic interaction of novel lactate dehydrogenase inhibitors with gemcitabine against pancreatic cancer cells in hypoxia. Br J Cancer.

[256] McEwan C, Owen J, Stride E, Fowley C, Nesbitt H, Cochrane D (2015). Oxygen carrying microbubbles for enhanced sonodynamic therapy of hypoxic tumours. J Control Release.

[257] Sheng Y, Nesbitt H, Callan B, Taylor MA, Love M, McHale AP (2017). Oxygen generating nanoparticles for improved photodynamic therapy of hypoxic tumours. J Control Release.

[258] Hu D, Chen Z, Sheng Z, Gao D, Yan F, Ma T (2018). A catalase-loaded hierarchical zeolite as an implantable nanocapsule for ultrasound-guided oxygen self-sufficient photodynamic therapy against pancreatic cancer. Nanoscale.

[259] Kang S, Gil YG, Min DH, Jang H (2020). Nonrecurring Circuit Nanozymatic Enhancement of Hypoxic Pancreatic Cancer Phototherapy Using Speckled Ru-Te Hollow Nanorods. ACS Nano.

[260] Qian X, Zheng Y, Chen Y (2016). Micro/Nanoparticle-Augmented Sonodynamic Therapy (SDT): Breaking the Depth Shallow of Photoactivation. Adv Mater.

[261] Chen J, Luo H, Liu Y, Zhang W, Li H, Luo T (2017). Oxygen-Self-Produced Nanoplatform for Relieving Hypoxia and Breaking Resistance to Sonodynamic Treatment of Pancreatic Cancer. ACS Nano.

[262] Chen Y, Cairns R, Papandreou I, Koong A, Denko NC (2009). Oxygen consumption can regulate the growth of tumors, a new perspective on the Warburg effect. PLoS ONE.

